# Structural basis for CSPG4 as a receptor for TcdB and a therapeutic target in *Clostridioides difficile* infection

**DOI:** 10.1038/s41467-021-23878-3

**Published:** 2021-06-18

**Authors:** Peng Chen, Ji Zeng, Zheng Liu, Hatim Thaker, Siyu Wang, Songhai Tian, Jie Zhang, Liang Tao, Craig B. Gutierrez, Li Xing, Ralf Gerhard, Lan Huang, Min Dong, Rongsheng Jin

**Affiliations:** 1 Department of Physiology and Biophysics, University of California, Irvine, CA USA; 2 Department of Urology, Boston Children’s Hospital, Harvard Medical School, Boston, MA USA; 3 Department of Microbiology, Harvard Medical School, Boston, MA USA; 4 Department of Surgery, Harvard Medical School, Boston, MA USA; 5 Department of Gastrointestinal, Colorectal and Anal Surgery, China-Japan Union Hospital of Jilin University, Changchun, China; 6 Center for Infectious Disease Research, Key Laboratory of Structural Biology of Zhejiang Province, School of Life Sciences, Westlake University, Hangzhou, Zhejiang China; 7 Institute of Basic Medical Sciences, Westlake Institute for Advanced Study, Hangzhou, Zhejiang China; 8 UC Irvine Materials Research Institute (IMRI), University of California, Irvine, CA USA; 9 Institute of Toxicology, Hannover Medical School, Hannover, Germany

**Keywords:** Bacterial toxins, Cryoelectron microscopy

## Abstract

*C. difficile* is a major cause of antibiotic-associated gastrointestinal infections. Two *C. difficile* exotoxins (TcdA and TcdB) are major virulence factors associated with these infections, and chondroitin sulfate proteoglycan 4 (CSPG4) is a potential receptor for TcdB, but its pathophysiological relevance and the molecular details that govern recognition remain unknown. Here, we determine the cryo-EM structure of a TcdB–CSPG4 complex, revealing a unique binding site spatially composed of multiple discontinuous regions across TcdB. Mutations that selectively disrupt CSPG4 binding reduce TcdB toxicity in mice, while CSPG4-knockout mice show reduced damage to colonic tissues during *C. difficile* infections. We further show that bezlotoxumab, the only FDA approved anti-TcdB antibody, blocks CSPG4 binding via an allosteric mechanism, but it displays low neutralizing potency on many TcdB variants from epidemic hypervirulent strains due to sequence variations in its epitopes. In contrast, a CSPG4-mimicking decoy neutralizes major TcdB variants, suggesting a strategy to develop broad-spectrum therapeutics against TcdB.

## Abstract (web summary)

Chondroitin sulfate proteoglycan 4 (CSPG4) is a potential receptor for *C. difficile* toxin B (TcdB) during *C. difficile* infections (CDIs). Here, the cryo-EM structure of a TcdB–CSPG4 complex and CDI mouse models offer insights into CSPG4 role in CDIs and suggest a therapeutic strategy targeting TcdB.

## Introduction

*Clostridioides difficile* (formerly *Clostridium difficile*, or *C. difficile)* is a Gram-positive, spore-forming anaerobic bacterium. With estimated ~223,900 infections, 12,800 deaths, and $1 billion healthcare cost in the US in 2017, *C. difficile* infection (CDI) is the most frequent cause of healthcare-acquired gastrointestinal infections and death in developed countries^1,2^. There is also increasing frequency of community-associated infections in recent years^1,3,4^. Two homologous *C. difficile* exotoxins (TcdA and TcdB) are the major virulence factors. Among them, TcdB alone is capable of causing the full-spectrum of diseases associated with CDI in humans, and pathogenic TcdA^−^TcdB^+^ strains have been isolated in clinic^5–7^. The key role of TcdB in CDI is further confirmed by the finding that an FDA-approved anti-TcdB monoclonal antibody (bezlotoxumab) reduced CDI recurrence in humans^8,9^.

TcdB (~270 kDa) is composed of four structural modules: a N-terminal glucosyltransferase domain (GTD), followed by a cysteine protease domain (CPD), an intermingled membrane translocation delivery domain and receptor-binding domain (DRBD), and a large C-terminal combined repetitive oligopeptides domain (CROPs)^10^. It is well accepted that the DRBD and CROPs are responsible for receptor recognition, and the two enzymatic domains GTD and CPD are delivered to the cytosol where the GTD glucosylates small GTPases of the Rho family, leading to actin cytoskeleton disruption and cell death^5,11^. It is worth noting that a unique hinge region located between the DRBD and CROPs is essential for toxicity, which serves as a critical structural linchpin to mediate structural communications among all four domains of TcdB^10,12,13^.

Beyond its complex 3D structure, TcdB has greatly diversified throughout its entire primary sequence up to 11% during evolution^14–16^. For example, many hypervirulent fluoroquinolone-resistant lineages such as BI/NAP1/027 strains, which emerged in North America with major outbreaks in early 2000s, express a variant of TcdB (designated TcdB2) that is ~8% sequence variation from the endemic TcdB (designated TcdB1)^15–18^. The sequence variations have impacts on TcdB activity and pathogenicity as evidence by the observations that bezlotoxumab showed ~200-fold lower potency on neutralizing TcdB2 than TcdB1^19,20^. Therefore, the complexity of TcdB variation has posed significant challenges for developing effective therapeutic antibodies, vaccines, and diagnostic assays with sufficient broadness.

Another major concern arises from the observation that TcdB variants may have changed their strategies to recognize host receptors for cell entry. Recent studies have identified the Wnt receptor frizzled proteins (FZDs) and chondroitin sulfate proteoglycan 4 (CSPG4, also known as NG2 in rodents) as two major candidate receptors for TcdB^21–24^. CSPG4 is a single transmembrane domain protein conserved across evolution, with no apparent redundant isoforms in humans. Unlike FZDs that are expressed in the colonic epithelium, CSPG4 is highly expressed in many immature progenitor cells such as oligodendrocyte progenitor cell and mesenchymal stem cells^25,26^. While its function remains to be fully established, it has been shown to promote cell proliferation, adhesion, migration, as well as mediate binding of many growth factors such as basic fibroblast growth factor and integrin. TcdB1 could bind FZDs and CSPG4 simultaneously, indicating that FZDs and CSPG4 are recognized by distinct regions of TcdB^21,27^. However, many clinically important TcdB variants, represented by TcdB2, bind CSPG4 but not FZDs, because they have residue substitutions in the FZD-binding site that abolish their binding to FZDs^14–16,27–29^. Moreover, the therapeutic antibody bezlotoxumab reduces binding of TcdB1 to CSPG4 in vitro^30^, suggesting CSPG4 may contribute to TcdB pathogenesis in humans. These findings suggest that CSPG4 could be a broad-spectrum receptor for diverse TcdB variants and a promising therapeutic target in CDI.

Here, we determine the cryogenic electron microscopy (cryo-EM) structure of a TcdB1–CSPG4 complex and identify a unique composite CSPG4-binding interface in TcdB, which involves residues scattering across multiple TcdB domains including its CPD. These CSPG4-binding residues are highly conserved across most TcdB variants known to date, and a rationally designed CSPG4-mimicking decoy potently inhibits both TcdB1 and TcdB2. We further show that bezlotoxumab disrupts this CSPG4-binding site via an allosteric manner, but its epitopes are susceptible to escaping mutations in TcdB. These studies establish the essential role of CSPG4 as a key TcdB receptor and reveal strategies for developing broad-spectrum therapeutics for the treatment of CDI.

## Results

### Structure determination of the TcdB–CSPG4 complex by cryo-EM

**Fig. 1:**
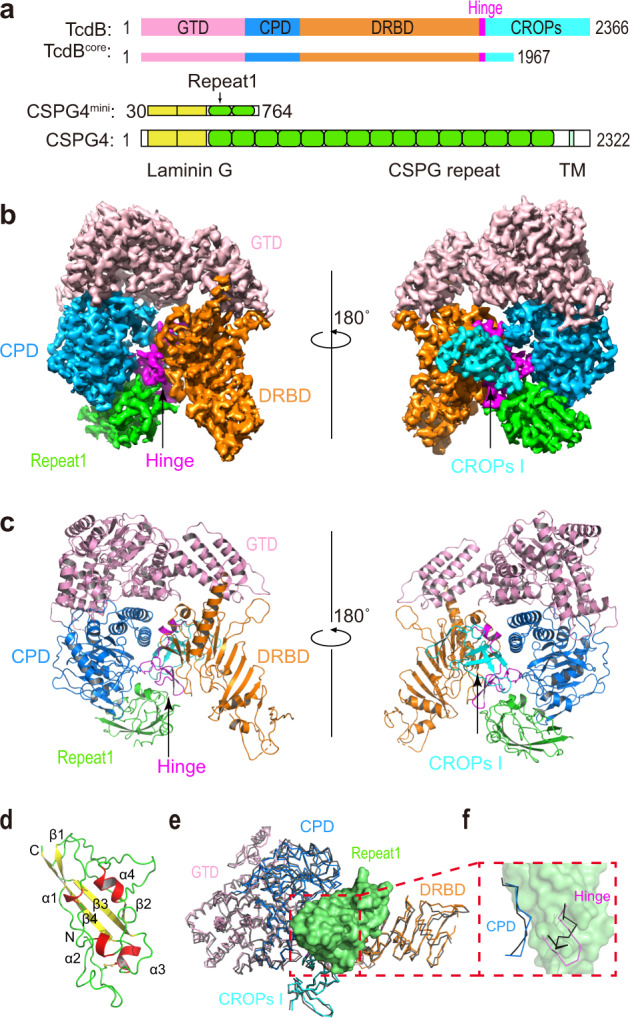
Overall structure of the TcdB–CSPG4 complex. **a** Schematic diagrams showing the domain structures of TcdB and CSPG4, as well as the domain boundaries for TcdB^core^ and CSPG4^mini^ used for cryo-EM studies. GTD: glucosyltransferase domain, CPD: cysteine protease domain, DRBD: delivery and receptor-binding domain, CROPs: combined repetitive oligopeptides domain, Hinge: a key fragment between the DRBD and CROPs that mediates structural communications among all four domains of TcdB. CSPG4 is composed of two predicted laminin G domains, 15 CSPG repeats, a transmembrane domain (TM), and a cytosolic region. **b** The 3.17 Å resolution cryo-EM map of the TcdB^core^–Repeat1 complex segmented and colored as shown in **a**. **c** Cartoon representation of the structure of the TcdB^core^–Repeat1 complex that is shown in similar orientations and color schemes as that in **b**. **d** The structure of Repeat1 of CSPG4 with the disulfide bond shown as sticks. **e** The structure of the TcdB^core^–Repeat1 complex was superimposed to TcdB holotoxin (PDB: 6OQ5). The Repeat1-bound TcdB is colored as shown in **a** and the unliganded TcdB is colored black with its CROPs II–IV omitted for clarity. The TcdB-bound Repeat1 is shown as a green surface model. **f** Repeat1 triggers local structural changes in the CPD and hinge of TcdB upon binding. For clarity, only residues 569–577 in the CPD and residues 1803–1812 in the hinge are shown in the context of Repeat1 (green surface).

CSPG4 is a large highly glycosylated single transmembrane protein (~251 kDa). Its extracellular domain was predicted to contain a signal peptide, two laminin G motifs, and 15 consecutive CSPG repeats^25^ (Fig. 1a). Our initial efforts using the recombinant full extracellular domain of human CSPG4 (residues 30–2204, referred to as CSPG4^ECD^) and TcdB1 holotoxin (VPI10463 strain) were hampered by the structural flexibility of TcdB and CSPG4^10,25^. We then sought to first define the interacting domains within TcdB1 and CSPG4^ECD^ employing cross-linking mass spectrometry (XL-MS) using the MS-cleavable cross-linker dihydrazide sulfoxide (DHSO)^31^ (Supplementary Fig. 1a, b and Source Data). When forming a complex, acidic residues in TcdB1 and CSPG4^ECD^ that have Cα-Cα distances within 35 Å can be cross-linked by DHSO, and the resulting cross-linked peptides could be identified using multistage mass spectrometry (MS^*n*^)^32,33^.

We identified a total of 263 unique DHSO cross-linked peptides of the TcdB1–CSPG4^ECD^ complex (Supplementary Table 1), representing 18 inter-protein and 245 intra-protein (167 in TcdB1 and 78 in CSPG4^ECD^) cross-links. The intra-molecular cross-links in TcdB1 show good correlations with the crystal structure of TcdB1 holotoxin that we recently reported^10^. Fourteen pairs of the inter-protein cross-links were mapped to the first predicted CSPG repeat and the CPD and the N-terminus of DRBD of TcdB, indicating direct interactions between them (Supplementary Fig. 1c). The rest four pairs of cross-links suggested that the laminin G domains of CSPG4 may adopt flexible conformations and could transiently move within ~35 Å of the CPD or DRBD of TcdB, because the same residues (e.g., E92/E93) in this region of CSPG4 could be cross-linked to amino acids on the CPD and DRBD of TcdB that are 97 Å away from each other (Supplementary Table 1). Guided by the XL-MS results, we analyzed interactions between a number of fragments of TcdB1 and CSPG4 and their biochemical behaviors, and narrowed down a fragment of TcdB1 (residues 1–1967, referred to as TcdB^core^) that contains the GTD, CPD, DRBD, and the first unit of CROPs (termed CROPs I), which could robustly bind to an N-terminal CSPG4 fragment composed of two laminin G motifs and first two CSPG repeats (residues 30–764, referred to as CSPG4^mini^) (Fig. 1a and Supplementary Table 2).

We successfully obtained a stable complex composed of TcdB^core^ and CSPG4^mini^, which was used for cryo-EM study (Supplementary Fig. 2a–d). The preliminary data analysis yielded a 3.4 Å resolution structure for the TcdB^core^–CSPG4^mini^ complex, which revealed that CSPG4^mini^ binds to a groove in TcdB that is surrounded by the CPD, DRBD, hinge, and CROPs I (Supplementary Fig. 2e, f), which is consistent with our XL-MS studies. 3D variability analysis indicated that the distal region in the DRBD of TcdB and the N-terminal two laminin G motifs of CSPG4^mini^ were highly flexible, which hindered us from obtaining a high-resolution map for de novo model building. Notably, these flexible regions in TcdB and CSPG4 were outside the complex interface. Therefore, we could improve the resolution by using a smaller box size during particle picking to focus on the TcdB–CSPG4 interface. With a focused refinement, we were able to further improve the density map to 3.17 Å resolution that allowed de novo model building for CSPG4, while the TcdB structure was built using the crystal structure of TcdB holotoxin as a model^10^ (Fig. 1b, c and Supplementary Fig. 2g, h). Structure determination statistics and representative density maps for the protein complex were shown in Supplementary Table 3 and Supplementary Fig. 3.

### TcdB1 and TcdB2 use a conserved composite binding site for CSPG4

**Fig. 2:**
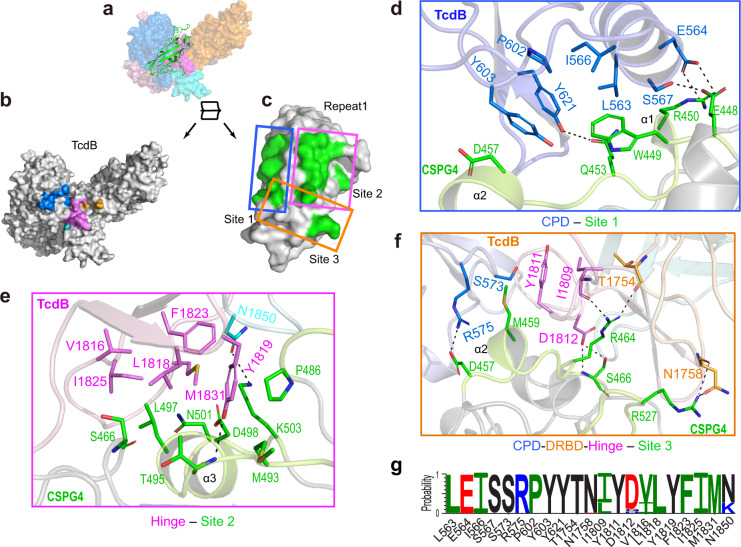
TcdB recognizes CSPG4 using a composite binding site involving multiple domains. **a** CSPG4 Repeat1 binds at a groove formed by the CPD, DRBD, hinge, and CROPs I. TcdB^core^ and Repeat1 are shown as a surface and a cartoon representation, respectively. **b**, **c** An open-book view of the TcdB^core^–Repeat1 interface. CSPG4-binding residues in the CPD, DRBD, hinge, and CROPs are colored blue, orange, purple, and cyan, respectively (**b**). The amino acids in Repeat1 that constitute the three TcdB-binding subsites are colored green and outlined in blue, purple, and orange boxes (**c**), while their detailed interactions with TcdB are further illustrated in **d**–**f**. **d**–**f** Close-up views of the TcdB–CSPG4 interface with interacting amino acids shown in stick models. Residues in the CPD, DRBD, hinge, and CROPs I of TcdB are colored blue, orange, purple, and cyan, respectively, while Repeat1 resides are colored green. **g** Graphical representations of sequence conservation of CSPG4-binding residues in TcdB. The height of symbols at each position indicates the relative frequency of each amino acid at that position based on analyses of 206 unique TcdB variants.

The structure of the TcdB–CSPG4 complex reveals that the first CSPG repeat of CSPG4 (termed Repeat1, residues 410–551) is mainly responsible for TcdB binding, while the rest of CSPG4 pointing away from the toxin (Supplementary Fig. 2f). Repeat1 has a compact structure consisting of a four-strand β sheet and 4 short α helices, which are connected by intermittent loops and stabilized by a disulfide bridge (Fig. 1d). Despite its small size, Repeat1 directly interacts with many amino acids that are dispersed across over 1300 residues on the primary sequence of TcdB, including the CPD, DRBD, hinge, and CROPs (Fig. 1b, c). All these TcdB residues converge spatially to form a composite binding site for Repeat1 involving an extensive interaction network and burying a large molecular interface between them (∼2715.5 Å^2^) (Fig. 2a–c). This unusually complex binding mode, especially the involvement of the CPD, is unexpected, because it was previously believed that the receptor binding of TcdB is carried out by the DRBD and the CROPs^5,21,30^.

More detailed structural analysis showed that the TcdB-binding surface in Repeat1 could be divided into three subsites (Fig. 2c). The site-1 of Repeat1 (residues 448–457) binds to the CPD via hydrogen bonds, charge–charge interaction, as well as a large patch of hydrophobic interactions (Fig. 2d and Supplementary Table 4). The site-2 of Repeat1 (residues 466–503) binds to the hinge of TcdB involving mainly hydrophobic interaction and two hydrogen bonds, and also interacts with the CROPs I with a hydrogen bond (Fig. 2e and Supplementary Table 4). The site-3 of Repeat1 is composed of two separated areas including residues 457–466 and an additional residue (R527) in a nearby loop. It binds to a composite interface in TcdB, which is composed of residues in the CPD, DRBD, and hinge (Fig. 2f and Supplementary Table 4). CSPG4 is predicted to have 15 N-linked glycosylation site with one in Repeat1 (N427) and a single chondroitin sulfate modification at S995^25^. We did not observe density for the N427 glycan and it remains to be determined whether these glycans in CSPG4 may contribute to TcdB recognition.

The overall structure of the CSPG4-bound TcdB^core^ is similar to the crystal structure of TcdB holotoxin with a root mean square deviation between comparable Cα atoms about 1.06 Å (Fig. 1e)^10^. Nevertheless, CSPG4 binding triggers local structural changes in TcdB involving residues 1803–1812 in the hinge and 569–577 in the CPD (Fig. 1f). It is worth noting that the hinge is located at a strategic site in TcdB communicating with all four major domains, and the CROPs of TcdB adopts dynamic conformations relative to the rest of the toxin^10^. Therefore, conformational changes in TcdB could affect the structure of the hinge and the configuration of the CSPG4-binding site that would subsequently influence CSPG4 binding, while CSPG4 binding could in turn modulate TcdB structure.

We further carried out real-time analysis of the kinetics of TcdB–CSPG4 interactions using bio-layer interferometry (BLI). For this study, we first designed a recombinant CSPG4 Repeat1 that is fused to the N-terminus of the Fc fragment of a human immunoglobulin G1 (Repeat1-Fc). Based on the structural modeling, the Fc fragment in Repeat1-Fc does not interfere with TcdB binding, and provide a convenient way for immobilization of Repeat1-Fc to the biosensors. We found that TcdB1 recognized Repeat1-Fc with a high affinity (dissociation constant, *K*_d_ ~15.2 nM) (Supplementary Fig. 4a). Notably, Repeat1-Fc binds to TcdB with a relatively slow on-rate (*k*_on_ ~7.06 × 10^3^ M^−1^ s^−1^), which is likely due to organization of multiple structural units in TcdB to form the composite binding site for CSPG4. Nevertheless, once Repeat1 is engaged with TcdB, the complex is very stable as evidence by their slow binding off-rate (*k*_off_ ~1.08 × 10^−4^ s^−1^).

Since TcdB1 and TcdB2 have different primary sequences and pathogenicity, we carried out structure-based sequence analysis between them focusing on the CSPG4-binding site. Remarkably, the key amino acids consisting the composite CSPG4-binding site are nearly identical between TcdB1 and TcdB2, even though these residues scatter across multiple TcdB domains (Fig. 2g). It is worth noting that the hinge region has large sequence variations among TcdB isoforms, and the hypervariable sequences in this region are believed to contribute to differences in toxicity and antigenicity of TcdB2 and other variants^12^. But the CSPG4-binding residues in the hinge are conserved between TcdB1 and TcdB2 except for two conservative substitutions of I1809^TcdB1^ with L1809^TcdB2^ and V1816^TcdB1^ with I1816^TcdB2^. The only other difference is N1850^TcdB1^ in the CROPs I that forms a hydrogen bond with K503 of CSPG4 is replaced with K1850^TcdB2^. Nevertheless, our BLI binding studies showed that TcdB2 binds to Repeat1-Fc with a high affinity that is even slightly better than TcdB1 (*K*_d_ ~5.4 nM, *k*_on_ ~8.34 × 10^3^ M^−1^ s^−1^, *k*_off_ ~4.63 × 10^−5^ s^−^^1^) (Supplementary Fig. 4b). Therefore, the three residue substitutions in the CSPG4-binding site are well tolerated in TcdB2. These data demonstrate that the CSPG4-binding mode is conserved between TcdB1 and TcdB2.

### Site-specific mutagenesis to validate TcdB–CSPG4 interactions

**Fig. 3:**
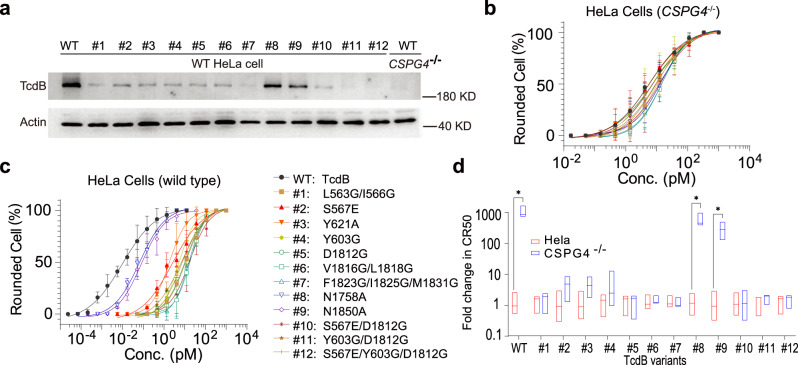
Structure-based mutagenesis analyses of the interactions between TcdB and CSPG4. **a** The indicated TcdB mutants were tested for binding to cells. Purified WT and mutated TcdB (10 nM) were incubated with WT or CSPG4^−/−^ HeLa cells. Cells were washed three times by PBS, harvested, and cell lysates were analyzed by immunoblot detecting TcdB. Actin served as a loading control. The sensitivity of CSPG4^−/−^ (**b**) and WT (**c**) HeLa cells to mutated TcdB was examined using the standard cytopathic cell-rounding assay. Error bars indicate mean ± sd (*n* = 3 biologically independent experiments). **d** The ratios of CR_50_ values on CSPG4^−/−^ vs. WT HeLa cells from **b** and **c** were calculated and plotted, reflecting the fold-of-change in reduction of toxicity on CSPG4^−/−^ cells compared with WT cells. *n* = 3 for all groups. The upper and lower bounds of boxes indicate the maximum and minimum values of each group. The middle lines indicate the median values of each group. *p* values by *t*-test: *p ≤ 0.05.

We next carried out structure-guided mutagenesis of TcdB1 and CSPG4 to validate the binding interface and to define loss-of-function mutations in TcdB that could selectively abolish CSPG4 binding. We designed and characterized nine mutations of TcdB1 holotoxin, where the key CSPG4-binding residues in the CPD (L563G/I566G, S567E, Y621A, or Y603G), the hinge (D1812G, V1816G/L1818G, or F1823G/I1825G/M1831G), the DRBD (N1758A), or the CROPs I (N1850A) were mutated (Supplementary Fig. 5). These TcdB1 mutants showed reduced binding to HeLa cells expressing endogenous CSPG4^21^ (Fig. 3a). TcdB-N1758A and N1850A showed the least reduction of binding, suggesting that these two mutations, located in the DRBD and the CROPs respectively, have relatively weaker impact on TcdB–CSPG4 interactions compared with mutations in the CPD or the hinge. We then designed three combinational mutations of TcdB to simultaneously disrupt the anchoring points for CSPG4 in both the CPD and the hinge, including S567E/D1812G, Y603G/D1812G, and S567E/Y603G/D1812G, and found them largely abolished binding of TcdB to cells. Similar results were confirmed using pull-down assays with Repeat1-Fc as the bait and TcdB variants as preys (Supplementary Fig. 6a). We also designed and characterized variants of CSPG4 Repeat1 that carried site-specific mutations in the TcdB-binding interface, including mutations in site-1 (R450G, E448A, W449G, W449D, Q453A, E448A/W449D, R450G/Q453A), site-2 (L497G, L497D, L497G/D498G), and site-3 (D457G, R464A/S466G) (Supplementary Fig. 7). These mutations effectively disrupted the binding of TcdB holotoxin to Repeat1 based on pull-down assays (Supplementary Fig. 6b).

We further examined how these TcdB mutations effect CSPG4-mediated cytopathic toxicity at functional levels using standard cell-rounding assays, where TcdB entry would inactivate Rho GTPases and cause the characteristic cell-rounding phenotype^34^. The concentration of TcdB that induces 50% of cells to be round is defined as cell-rounding 50 (CR_50_), which is utilized to compare the potency of TcdB variants on the wild-type (WT) HeLa cells that express both CSPG4 and FZDs or the CSPG4 knockout (KO) HeLa cells. As shown in Fig. 3b, all 12 mutant TcdB1 induced cell-rounding with potencies similar to TcdB1 on CSPG4 KO cells, demonstrating that these mutations were properly folded and did not affect FZD-mediated binding and entry of toxins. In contrast, these mutant toxins showed various reduced potencies on WT HeLa cells compared with TcdB1 (Fig. 3c). More specifically, WT TcdB1 showed over 600-fold reduced toxicity on CSPG4 KO cells compared with WT cells, while the toxicity of TcdB1 variants carrying L563G/I566G, D1812G, V1816G/L1818G, F1823G/I1825G/M1831G, and the three combinational mutations were similar on CSPG4 KO cells and WT cells (CR_50_ ratio ~1.1–1.3), demonstrating that these mutations effectively and selectively eliminated CSPG4-mediated toxicity on cells (Fig. 3d).

### CSPG4 is a physiologically relevant receptor in vivo

Given our extensive structural, in vitro, and ex vivo data demonstrating the role of CSPG4 as a TcdB receptor, we sought to determine the contribution of CSPG4 to TcdB1 and TcdB2 pathogenicity and its relationship with FZD in vivo using two complementary approaches that were custom designed for TcdB2 and TcdB1, respectively.

We first used a *C. difficile* mutant strain (M7404, *tcdA*^*−*^) that only expresses TcdB2^35^ to directly assess the contribution of CSPG4 in vivo since TcdB2 does not bind to FZDs. We carried out infection experiments in mouse models based on established protocols (antibiotic treatment followed with gavage feeding of 1 × 10^5^ *C. difficile* spores) (Supplementary Fig. 8a) to compare pathological development in WT vs. CSPG4 KO mice^36^. All mice developed CDI symptoms including diarrhea and body weight loss, but it was less severe in CSPG4 KO mice than the WT mice in general. In addition, infection led to 100% moribundity of WT mice by 48 h, whereas only 50% of CSPG4 KO mice reached moribundity (Supplementary Fig. 8b).

**Fig. 4:**
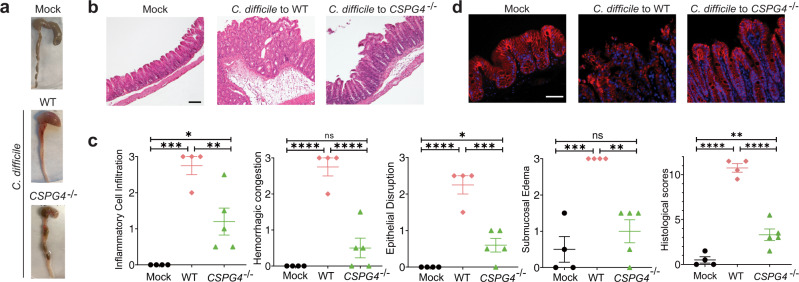
CSPG4 is a physiological relevant cellular receptor for TcdB in vivo. **a** Three groups of infection experiment were performed: mock to WT (*n* = 4); M7404, *tcdA*^*−*^ to WT mice (*n* = 8); and M7404, *tcdA*^*−*^ to CSPG4^−/−^ mice (*n* = 9). The representative cecum and colon of infected mice that were harvested at 48 h. The harvested cecum was processed with hematoxylin and eosin staining (scale bar represents 100 µm, mock *n* = 4, *C. difficile* to WT *n* = 4, *C. difficile* to CSPG4^−/−^ *n* = 5) (**b**), scored based on inflammatory cell infiltration, hemorrhagic congestion, epithelial disruption, and submucosal edema (**c**), and subjected to immunofluorescence staining by epithelial cell junction marker Claudin-3 (scale bar represents 50 µm, mock *n* = 3, *C. difficile* to WT *n* = 3, *C. difficile* to CSPG4^−/−^ *n* = 3) (**d**). In **c**, error bars indicate mean ± SEM (mock *n* = 4, *C. difficile* to WT *n* = 4, *C. difficile* to CSPG4^−/−^ *n* = 5). *p* values were calculated by  post hoc analysis of a one-way ANOVA using Holm-Sidak’s test for multiple comparisons: ****p ≤ 0.0001, ***p ≤ 0.001, **p ≤ 0.01, *p ≤ 0.05. The exact *p* values are presented in the accompanying source data.

We next carried out histological analysis of cecum and colon tissues. There was bloody fluid accumulation in tissues dissected from WT mice after infection, whereas there was much less fluid accumulation in tissues from CSPG4 KO mice (Fig. 4a). We further carried out histological analysis with paraffin-embedded cecum tissue sections (Fig. 4b), which were scored based on disruption of the epithelium, hemorrhagic congestion, submucosal edema, and inflammatory cell infiltration, on a scale of 0–3 (normal, mild, moderate, or severe, Fig. 4c). Infection induced extensive disruption of the epithelium and inflammatory cell infiltration, as well as severe hemorrhagic congestion and mucosal edema on WT mice (Fig. 4c). CSPG4 KO mice showed only moderate levels of epithelial damage and inflammatory cell infiltration, and mild to no hemorrhagic congestion and submucosal edema (Fig. 4b, c). Furthermore, TcdB2 induced extensive loss of tight junction in the cecum epithelium from WT mice based on immunofluorescence staining for a tight junction marker Claudin-3, while it was largely intact in CSPG4 KO mice (Fig. 4d). We observed similar results when we carried out infection experiments using a ten-fold lower dose of *C. difficile* spores (1 × 10^4^), which did not result in death of mice and thus allowed us to harvest cecum tissues 90 h after infection (Supplementary Fig. 8c–e). Analysis of feces indicated similar levels of *C. difficile* colonization and toxin titer in WT and CSPG4 KO mice (Supplementary Fig. 8c). Taken together, these results demonstrated that CSPG4 is a major receptor for the epidemic TcdB2 in vivo. The residual toxicity of TcdB2 in CSPG4 KO mice indicates that TcdB2 may have unknown low affinity receptor(s) that remains to be further evaluated.

**Fig. 5:**
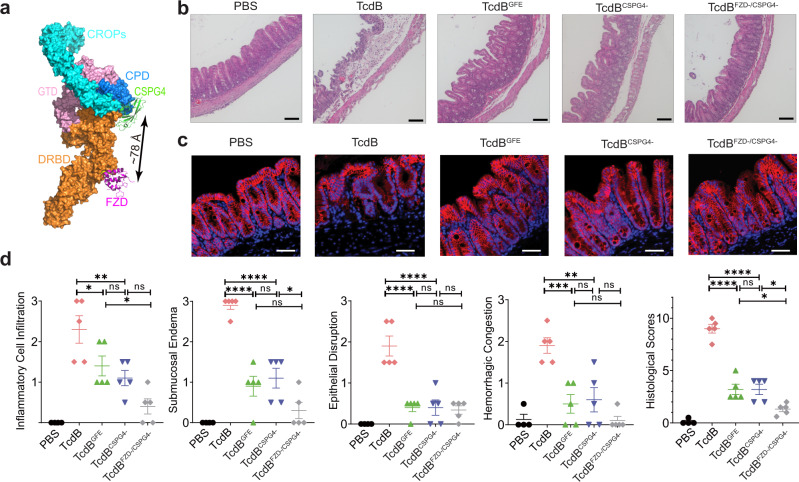
Mutations selectively abolishing CSPG4 or FZD binding reduce toxicity of TcdB on cecum tissues. **a** A structural model of TcdB holotoxin with CSPG4 and FZD bound at two independent sites. The model is built based on superposition of the structures of TcdB1 holotoxin (PDB: 6OQ5), the TcdB–FZD complex (PDB: 6C0B), and the TcdB–CSPG4 complex (this work). **b**–**d** The indicated TcdB mutants or the control PBS was injected into the cecum of CD1 mice in vivo. The cecum tissues were harvested 6 h later and subjected to histological analysis with representative images (scale bars represent 100 µm, PBS *n* = 4, TcdB *n* = 5, TcdB^GFE^ *n* = 5, and TcdB^FZD−/CSPG4−^ *n* = 5, TcdB^CSPG4−^ *n* = 5) (**b**), immunostaining analysis for the tight junction marker Claudin-3 (scale bars represent 50 µm, PBS *n* = 3, TcdB *n* = 3, TcdB^GFE^ *n* = 3, and TcdB^FZD−/CSPG4−^ *n* = 3, TcdB^CSPG4−^ *n* = 3) (**c**), and pathological scores (error bars indicate mean ± SEM, PBS *n* = 4, TcdB *n* = 5, TcdB^GFE^ *n* = 5, and TcdB^FZD−/CSPG4−^ *n* = 5, TcdB^CSPG4−^ *n* = 5) (**d**). *p* values were calculated by post hoc analysis of a by one-way ANOVA using Holm-Sidak’s test for multiple comparisons: ****p ≤ 0.0001, ***p ≤ 0.001, **p ≤ 0.01, *p ≤ 0.05. The exact *p* values are presented in the accompanying source data.

TcdB1 can be simultaneously bound by CSPG4 and FZD as demonstrated by our cryo-EM structure of the TcdB–CSPG4 complex and the crystal structure of a TcdB–FZD complex, which was confirmed by a pull-down experiment (Supplementary Fig. 8f)^24^. The estimated distance between the centers of CSPG4- and FZD-binding sites in TcdB is about 78 Å, and the two receptors are located on the same side of TcdB, making them possible to simultaneously anchor to the plasma membrane (Fig. 5a). To investigate the relationship of these two receptors for TcdB1, we resorted to three structure-based rationally designed TcdB1 mutants as molecular tools, which carry site-specific mutations to selectively knockout its binding capacity to CSPG4, FZD, or both. Based on the mutagenesis studies described above, we chose to use TcdB^S567E/Y603G/D1812G^ as a representative CSPG4 binding deficient TcdB mutant (TcdB^CSPG4−^). We previously already developed a FZD-binding deficient TcdB variant that carries mutations in the FZD-binding site (TcdB^GFE^)^24^. Combining these two TcdB mutants, we generated a unique TcdB variant that is unable to recognize either CSPG4 or FZD (TcdB ^FZD−/CSPG4−^).

We analyzed the toxicity of these TcdB1 mutants in comparison with the WT toxin by directly inject them into the mouse cecum^24,37^. This method has the advantage of controlling precisely the amount of toxins and incubation time, in order to capture any differences among these toxins. We found that WT TcdB1 induced severe damage to cecum tissues, resulting in inflammatory cell infiltration, submucosal edema, epithelial disruption, hemorrhagic congestion, and disruption of tight junction (Fig. 5b–d and Supplementary Fig. 8g). Both TcdB^GFE^ and TcdB^CSPG4−^ showed greatly reduced potency, with no significant difference between them: both showed modest levels of inflammatory cell infiltration and submucosal edema, and mild to normal levels of disruption of epithelium, tight junction, and hemorrhagic congestion. TcdB^FZD−/CSPG4−^ showed further reduced toxicity, with minimal levels of disruption to cecum tissues under our assay conditions (Fig. 5d and Supplementary Fig. 8g). These results demonstrate that FZDs and CSPG4 act as independent receptors in TcdB1 pathogenesis in vivo.

### Bezlotoxumab disrupts CSPG4-binding site in an allosteric manner

**Fig. 6:**
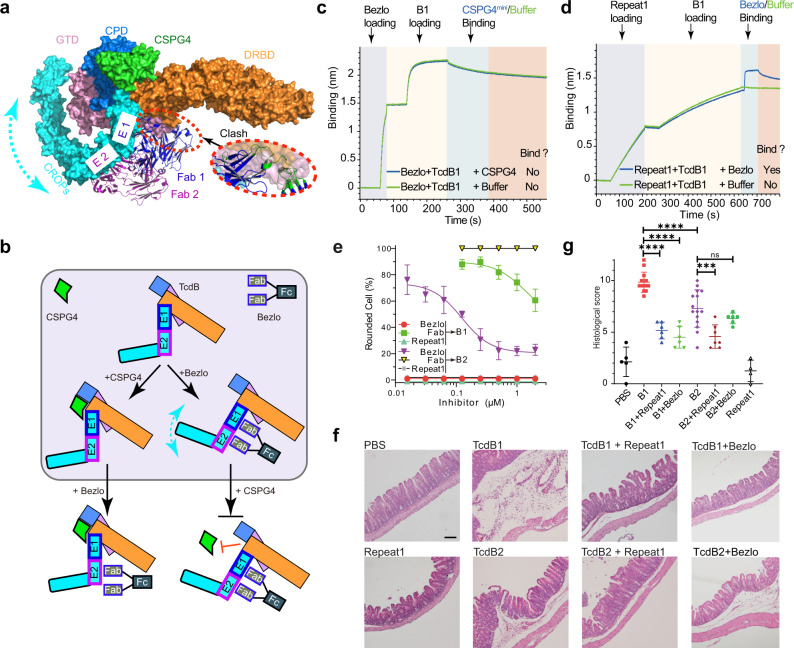
Bezlotoxumab competes with CSPG4 in an allosteric manner. **a** A structure model showing the binding of CSPG4 and bezlotoxumab (PDB: 4NP4) in TcdB holotoxin (PDB: 6OQ5). TcdB holotoxin and CSPG4 Repeat1 are showing as surface models with the GTD, CPD, DRBD, CROPs, and CSPG4 Repeat1 colored in pink, blue, orange, cyan, and green, respectively. The two Fab fragments of bezlotoxumab are shown as cartoon models and colored blue and purple. E1 and E2 indicate the epitope-1 and epitope-2 for bezlotoxumab in TcdB. A close-up view into the conflicting area between the Fab 1 bound at the E1 site and TcdB is shown in a red oval box, while the Fab residues that sterically clash with TcdB are colored in green. **b** A proposed model for allosteric interactions between CSPG4 and bezlotoxumab (Bezlo). **c** TcdB1 could not bind CSPG4^mini^ when it was prebound to the immobilized bezlotoxumab according to BLI assays. **d** Bezlotoxumab could still bind TcdB1 when it was prebound to the immobilized CSPG4 Repeat1. Sequential loading of different proteins to the biosensor is indicated by different background colors. **e** The protection effects of inhibitors against TcdB1 and TcdB2 were quantified by the cytopathic cell-rounding assay on HeLa cells. HeLa cells were incubated with TcdB1 (10 pM) or TcdB2 (100 pM) in the presence of serial-diluted bezlotoxumab (bezlo), its Fab (Fab), or Repeat1-Fc (Repeat1). Percentage of rounded cells are plotted by inhibitor concentrations at 6 h. Error bars indicate mean ± sd (*n* = 3 biologically independent experiments). **f**, **g** The protective effects of Repeat1-Fc and bezlotoxumab against TcdB1 and TcdB2 were examined in vivo using the cecum injection assay. TcdB1 (6 µg), TcdB2 (6 µg), TcdB1 or TcdB2 with Repeat1-Fc (30 µg) or bezlotoxumab (52 µg), Repeat1-Fc alone (30 µg), or the PBS control was injected into the cecum of CD1 mice in vivo. The cecum tissues were harvested 6 h later, and the representative H&E staining (scale bar represents 100 µm, PBS *n* = 5, B1 *n* = 13, B1 + Repeat1 *n* = 6, B1 + Bezlo *n* = 6, B2 *n* = 15, B2 + Repeat1 *n* = 7, B2 + Bezlo *n* = 6, Repeat1 *n* = 4) (**f**) and the histological scores (error bars indicate mean ± SEM, PBS *n* = 5, B1 *n* = 13, B1 + Repeat1 *n* = 6, B1 + Bezlo *n* = 6, B2 *n* = 15, B2 + Repeat1 *n* = 7, B2 + Bezlo *n* = 6, Repeat1 *n* = 4) (**g**) are shown. *p* values were calculated by post hoc analysis of a one-way ANOVA using Holm-Sidak’s multiple comparison test: ****p ≤ 0.0001, ***p ≤ 0.001, **p ≤ 0.01, *p ≤ 0.05. The exact *p* values are presented in the source data.

Bezlotoxumab is the only FDA-approved therapeutic antibody against TcdB, and a prior study suggested that bezlotoxumab reduced binding of TcdB to CSPG4 in vitro in immunoprecipitation assays^30^. However, bezlotoxumab recognizes two closely spaced homologous epitopes, epitope-1 and epitope-2, in the CROPs (Supplementary Fig. 9a)^20,38^, which is completely separated from the CSPG4-binding site, and therefore cannot directly compete with CSPG4. Since the prior structural studies were based on bezlotoxumab binding to a fragment of the CROPs, we generated a structural model of bezlotoxumab binding to TcdB holotoxin (Fig. 6a)^10^. We found that bezlotoxumab could bind to the epitope-2 without interfering the overall structure of TcdB, while its binding to epitope-1 would be hindered by the nearby GTD and DRBD. Therefore, bezlotoxumab has to force the CROPs domain to adopt a different orientation in order to gain access to epitope-1 and occupy both epitopes, which will benefit from the synergy between its two Fab arms (Fig. 6b). Since CSPG4 binds TcdB by simultaneously interacting with the CPD, DRBD, hinge, and CROPs, bezlotoxumab binding may reorient the CROPs relative to the rest of TcdB and compress the CSPG4-binding groove, thus preventing CSPG4 binding in an allosteric manner (Fig. 6b).

To verify this hypothesis, we examined the competition between bezlotoxumab and CSPG4 using BLI and pull-down assays. We found that when TcdB1 and TcdB2 were prebound with the immobilized bezlotoxumab, CSPG4 could not bind subsequently (Fig. 6c and Supplementary Fig. 9b, d). Meanwhile, the CSPG4-bound TcdB1 and TcdB2 could still bind bezlotoxumab, which is likely due to single-site antibody binding to epitope-2 (Fig. 6d and Supplementary Fig. 9c, e). To further understand how the single- vs. double-epitope binding modes effect bezlotoxumab’s activity, we examined the neutralization potency against TcdB1 for bezlotoxumab and its Fab fragment using the cell-rounding assay. When antibodies were preincubated with TcdB1 (10 pM) before adding to the culture medium, bezlotoxumab completely protected cells within 6 h at the lowest concentration tested (16 nM), but its Fab did not show any protection until the concentration reached 2 µM, which only reduced cell rounding by ~40% (Fig. 6e and Supplementary Fig. 10a, b). We believe this is due to the lack of synergy on TcdB binding between individual Fab molecules. These data consistently define a unique mechanism for bezlotoxumab at the molecular level, where it relies on synergistic binding to both epitopes in TcdB using its two Fab arms.

However, the need for bezlotoxumab to simultaneously occupy two epitopes in TcdB in order to be effective also increases its susceptibility to residue changes in TcdB variants. Epitope-1 and -2 in TcdB each consists of about 20 amino acids, and variations have been observed in many TcdB variants especially in epitope-1^16,20^ (Supplementary Fig. 10d, e). These amino acid substitutions in the bezlotoxumab-binding epitopes are believed to decrease the binding affinities and neutralization potencies of bezlotoxumab^20^. For example, the neutralization efficacy of bezlotoxumab on TcdB2 is ~200-fold lower than TcdB1^16,19^. Consistently, we found that bezlotoxumab showed a much lower potency in blocking TcdB2 on HeLa cells compared with TcdB1 in the cell-rounding assay, and its Fab failed to show any protection at the highest concentration tested (2 µM) (Fig. 6e and Supplementary Fig. 10b).

### A CSPG4 receptor decoy as a broad-spectrum TcdB inhibitor

As the CSPG4-binding site is conserved between TcdB1 and TcdB2, we envision that Repeat1 could be an effective CSPG4 decoy to block a broad range of TcdB. We thus evaluated the neutralization efficacies of Repeat1-Fc and bezlotoxumab against TcdB1 and TcdB2, which represent two largely diverged TcdB isoforms, using cell-rounding assays on HeLa cells. Repeat1-Fc at nM concentrations completely blocked both TcdB1 and TcdB2 within the 6-h incubation period, whereas bezlotoxumab only neutralized TcdB1, but not TcdB2 (Fig. 6e). Furthermore, bezlotoxumab at up to 2 µM failed to block TcdB1 or TcdB2 when incubation time was extended to 24 h, whereas Repeat1-Fc at the same concentration was still able to partially neutralize TcdB1 and TcdB2 and prevent ~40% cells from rounding (Supplementary Fig. 10c). These data demonstrate that Repeat1-Fc offers an enhanced protection against both TcdB1 and TcdB2 than bezlotoxumab.

We further evaluated Repeat1-Fc and bezlotoxumab for blocking TcdB1 and TcdB2 in vivo using the mouse cecum injection model. Briefly, TcdB1 or TcdB2 (6 µg) was preincubated with Repeat1-Fc (30 µg) or bezlotoxumab (52 μg), respectively, and the mixture was injected into the mouse cecum. The cecum tissues were dissected out for histological analysis 6 h later. As shown in Fig. 6f, g and Supplementary Fig. 10f, Repeat1-Fc was able to reduce overall damage to cecum tissues from both TcdB1- and TcdB2-treated mice, including less inflammatory cell infiltration, submucosal edema, hemorrhagic congestion, and epithelial disruption, while bezlotoxumab was only effective in reducing TcdB1 toxicity, but showed no effect on TcdB2 under the same assay conditions.

## Discussion

A major event in CDI epidemiology is the emergence of rapidly spreading hypervirulent strains globally in recent years, which together with a growing number of other strains produce diverse TcdB isoforms with sequence variations up to 11%^15,16,39^. Here, using complementary structural and functional studies, we identify the CSPG4-binding site in TcdB and demonstrate that CSPG4 is a common receptor for both TcdB1 and TcdB2, two largest families of TcdB isoforms responsible for over 70% of clinical isolates^16^. Furthermore, CSPG4 alone acts as a key protein receptor for the clinically important TcdB2 lacking the ability to bind FZDs. Our studies also reveal that bezlotoxumab acts via an allosteric mechanism to disrupt the CSPG4-binding site in TcdB, further demonstrating a key role of CSPG4 for TcdB pathogenesis in humans.

We find that CSPG4 and FZD bind to two distinct sites on TcdB, and both contribute to pathogenesis of TcdB1 in colonic tissues in vivo. Unlike FZDs that are expressed in the colonic epithelium, CSPG4 is highly expressed in sub-epithelial myofibroblast cells within the colonic tissues^21,40^, which are critical for maintenance, wound repair, and immune responses^41^. Therefore, our results suggest that myofibroblast cells could be a major target for TcdB. But how TcdB-mediated damages on myofibroblasts contribute to pathogenesis in CDI remains to be further explored. The possibilities of CSPG4 being expressed at low levels in colonic epithelium or transiently in colonic stem cell/progenitor cells or TcdB2 may recognize an unknown receptor in colonic epithelium remain to be further evaluated. As CSPG4 and FZD have different tissue distribution in vivo, the pathogenicity of a particular *C. difficile* strain could be partly attributed to the receptor-binding strategy of the TcdB isoform it produces, which opens up an interesting avenue for future research.

At the molecular level, Repeat1 of CSPG4 is gripped by TcdB at a groove jointly formed by the CPD, DRBD, hinge, and CROPs with each of them directly contributing to CSPG4 binding. The configuration of such a complex composite binding interface for CSPG4 involving multiple structural units in TcdB is unexpected, raising a need to revise our previous definition of receptor-binding domain for the family of large clostridial glucosylating toxins, which include TcdA and TcdB, *C. novyi* α-toxin (Tcnα), *C. sordellii* lethal, and hemorrhagic toxins (TcsL and TcsH), and *C. perfringens* toxin (TpeL)^42^. Furthermore, the need for a composite binding site for CSPG4 also exposes a vulnerability of TcdB, because it would be sensitive to conformational changes in the structurally flexible TcdB^10^. Indeed, bezlotoxumab binding to the CROPs would trigger TcdB conformational changes, which disrupt the CSPG4-binding site via an allosteric mechanism. But, paradoxically, the unique binding mode of bezlotoxumab on two epitopes in TcdB also exposes a weakness of bezlotoxumab itself. As TcdB is actively evolving, many TcdB mutations have been identified in the epitopes of bezlotoxumab, causing its notably decreased efficacy on many TcdB isoforms, let alone new TcdB mutants likely to emerge in the future^15,16,20,39^.

In contrast to the frequent sequence variations in the FZD- and the bezlotoxumab-binding sites, we found variations for only four CSPG4-binding residues among 206 unique TcdB variants available in DiffBase, the largest collection of TcdB variants up to date^16^. Our studies show that only one substitution at a residue equivalent to D1812^TcdB1^ (variation of 11.7%) could potentially affect CSPG4 binding, while variations at the other three residues, I1809^TcdB1^ (27.2%), V1816^TcdB1^ (27.7%), and N1850 ^TcdB1^ (16.7%), do not affect TcdB2 binding to CSPG4. We found 14 TcdB variant sequences in Diffbase containing D1812G, all of these sequences share ~98% identity with TcdB1 and have a well-preserved FZD-binding site, suggesting they could at least use FZDs as the receptors. Another ten sequences containing D1812H/K, which are more distant from TcdB1 with ~86–92% sequence identity and all are expected to already lose the ability to bind FZDs due to residue variations in their FZD-binding interfaces^24,28,43^. We suspect that a single substitution at D1812^TcdB1^ in these TcdB variants could be compensated by additional amino acid changes in the CSPG4-binding interface, although we cannot rule out the possibility that some of these TcdB variants might evolve to bind a yet unknown host receptor.

The high conservation of the CSPG4-binding site in diverse TcdB isoforms provides a unique therapeutic avenue for combating TcdB. We found that the recombinant Repeat1-Fc could potently inhibit toxicity of both TcdB1 and TcdB2 in a cell-based assay, which was superior than bezlotoxumab. Furthermore, this antibody-like decoy receptor protected mice from both TcdB1 and TcdB2 in vivo. Since we found that CSPG4 and FZDs act as two independent receptors for TcdB1, we envision that a bi-specific decoy receptor composed of both Repeat1 and the CRD of FZDs will further improve its potency against TcdB1-like toxins while maintaining its broad protection spectrum. Similar strategies have been exploited to combating viruses. For instance, a Fc-tagged bi-specific receptor decoy containing fragments of CD4 and CCR5 receptors neutralized a broad range of simian immunodeficient virus strains in vivo in primate models^44^. As an evolutionally conserved cellular receptor of TcdB, a CSPG4 decoy molecule would be difficult for TcdB to escape, since any mutations that disrupt toxin binding to the decoy would also disrupt binding to its native receptors. Taken together, our studies establish the mechanistic and structural foundation for the development of next generation therapeutics for the prevention and treatment of CDI, which will have broad activities across diverse *C. difficile* strains.

## Methods

### Cloning, expression, and purification of recombinant proteins

The genes of TcdB^core^ (residues 1–1967 of VPI10463 strain) and the full-length WT TcdB1 were cloned into modified pET22b and pET28a vectors, respectively, with a Twin-Strep tag followed by a human rhinovirus 3C protease cleavage site introduced to its N-terminus and a 6xHis tag to its C-terminus. Four point mutations (W102A/D286N/D288N/L543A) were introduced to the GTD of TcdB^core^ to eliminate the glucosyltransferase activity and thus its toxicity^45,46^, which was required by the biosafety regulation at PNCC. The gene of CSPG4^mini^ (residues 30–764) was cloned into a modified pcDNA vector with a human IL2 signal sequence (MYRMQLLSCIALSLALVTNS), a 9xHis tag, and a factor Xa–cleavage site added to its N-terminus. The gene of CSPG4 Repeat1 (residues 410–551) was cloned into a modified pcDNA vector with a human IgGk signal sequence (METDTLLLWVLLLWVPGSTG), an 8xHis tag, and a factor Xa–cleavage site added to its N-terminus, and a human Fc tag added to the C-terminus (Repeat1-Fc). The synthesized gene of the light chain of bezlotoxumab (Genewiz) and a His-tagged version of Repeat1 were cloned into the same vector with an 8xHis tag and a factor Xa–cleavage site added to its N-terminus. CSPG4 extracellular domain (residues 30–2204, referred to as CSPG4^ECD^) was cloned to the same vector with a C-terminal 7xHis tag. The synthesized genes of the complete heavy chain of bezlotoxumab and its V_H_-C_H_1 fragment (Genewiz) were cloned into the same vector, respectively, without any tag. Primers were listed in Supplementary Table 5. All TcdB and CSPG4 mutants were generated by two-step PCR and verified by DNA sequencing.

TcdB^core^, the Twin-Strep tagged full-length TcdB1, and all TcdB1 mutants were expressed in *E. coli* strain BL21-Star (DE3) (Invitrogen). Bacteria were cultured at 37 °C in LB medium containing kanamycin or ampicillin. The temperature was reduced to 18 °C when OD_600_ reached ~0.8. Expression was induced with 1 mM IPTG (isopropyl-b-D-thiogalactopyranoside) and continued at 18 °C overnight. The cells were harvested by centrifugation and stored at −80 °C until use. The recombinant full-length TcdB1 (VPI10463 strain) and TcdB2 (R20292 strain), which were used for affinity measurement and competition assays, were expressed in *Bacillus megaterium*^47^ and purified as described previously^10^.

The His-tagged proteins (TcdB^core^, Twin-Strep tagged full-length TcdB1, and TcdB1 mutants) were purified using Ni^2+^-NTA (nitrilotriacetic acid, Qiagen) affinity resins in a buffer containing 50 mM Tris, pH 8.0, 400 mM NaCl, and 40 mM imidazole. The proteins were eluted with a high-imidazole buffer (50 mM Tris, pH 8.0, 400 mM NaCl, and 300 mM imidazole) and then dialyzed at 4 °C against a buffer containing 20 mM HEPES, pH 7.5, and 150 mM NaCl. The Twin-Strep tagged TcdB^core^, TcdB1, and its variants were further purified using Strep-Tactin resins (IBA Lifesciences).

The His-tagged CSPG4^mini^, CSPG4^ECD^, Repeat1, Repeat1-Fc, and its mutants were expressed and secreted from FreeStyle HEK 293 cells (Thermo Fisher) by PEI-mediated transient transfection. Proteins were purified directly from cell culture medium using Ni^2+^-NTA resins, which were then eluted with a buffer containing 50 mM Tris, pH 8.0, 400 mM NaCl, 3 mM CaCl_2_, and 300 mM imidazole. Bezlotoxumab and its Fab were expressed by co-transfection of the light chain and the heavy chain, and the secreted proteins were purified via the His-tag on the light chain using Ni^2+^-NTA resins and the aforementioned buffer. CSPG4^mini^ was further purified by Superdex-200 size-exclusion chromatography using a buffer containing 20 mM HEPES, pH 7.5, 3 mM CaCl_2_, and 150 mM NaCl. To prepare the TcdB^core^–CSPG4^mini^ complex, the purified TcdB^core^ was first bound to Strep-Tactin resins for 3–4 h and the unbound TcdB^core^ was washed away using a buffer containing 20 mM HEPES, pH 7.5, 3 mM CaCl_2_, and 150 mM NaCl. The TcdB-bound resins were then mixed with a four-fold molar excess of the purified CSPG4^mini^ for 3–4 h. After the unbound CSPG4^mini^ was washed away, the protein complex was eluted by a buffer containing 20 mM HEPES, pH 7.5, 3 mM CaCl_2_, 50 mM D-biotin, and 150 mM NaCl and then dialyzed at 4 °C against a buffer containing 20 mM HEPES, pH 7.5, 3 mM CaCl_2_, and 150 mM NaCl. The TcdB–CSPG4^ECD^ complex were assembled using a similar strategy. The protein complexes were concentrated and stored at −80 °C until use.

### DHSO cross-linking of TcdB–CSPG4^ECD^

The purified TcdB–CSPG4^ECD^ complex (35 μl, 5 μM) was cross-linked with 65 mM DHSO and 65 mM 4-(4,6-Dimethoxy-1,3,5-triazin-2-yl)-4-methylmorpholinium chloride (DMTMM) in PBS (pH 7.4) for 1 h at room temperature using a protocol as previously described^48,49^. The resulting cross-linked products were subjected to enzymatic digestion using a FASP protocol^50^. Briefly, cross-linked proteins were transferred into Millipore Microcon Ultracel PL-30 (30-kDa filters), reduced/alkylated, and digested with Lys-C/trypsin as described^32^. The resulting digests were desalted and fractionated by peptide SEC^49^. The fractions containing DHSO cross-linked peptides were collected for subsequent LC MS^*n*^ analysis. Three biological replicates were performed to obtain highly reproducible cross-link data.

### LC MS^*n*^ analysis of DHSO cross-linked peptides

LC MS^*n*^ analysis was performed using a Thermo Scientific Dionex UltiMate 3000 system online coupled with an Orbitrap Fusion Lumos mass spectrometer. A 50 cm × 75 μm Acclaim PepMap C18 column was used to separate peptides over a gradient of 1–25% ACN in 106 min at a flow rate of 300 nl/min. Two different types of acquisition methods were utilized to maximize the identification of DHSO cross-linked peptides: (1) top four data-dependent MS^3^ and (2) targeted MS^3^ acquisition optimized for capturing DHSO cross-linked peptides by utilizing the mass difference between characteristic MS^2^ fragment ions of DHSO cross-linked peptides (*α* − *β*) (that Δ = *α*_T_ − *α*_A_ = *β*_T_ − *β*_A_ = 31.9721 Da)^51^.

### Data analysis and identification of DHSO cross-linked peptides

MS^*n*^ data extraction and analysis were performed as previously described^31,32^. MS^3^ data were subjected to Protein Prospector (v.5.19.1) for database searching, using Batch-Tag against a custom database containing nine protein entries concatenated with its random version. The mass tolerances were set as ±20 ppm and 0.6 Da for parent and fragment ions, respectively. Trypsin was set as the enzyme with three maximum missed cleavages allowed. Cysteine carbamidomethylation was set as a fixed modification. Variable modifications included protein N-terminal acetylation, methionine oxidation, and N-terminal conversion of glutamine to pyroglutamic acid. Additionally, three defined modifications on glutamic and aspartic acids were chosen, which included alkene (C_3_H_4_N_2_; +68 Da), sulfenic acid (C_3_H_6_N_2_SO; +118 Da), and thiol (C_3_H_4_N_2_S; +100 Da), representing cross-linker fragment moieties. Only a maximum of four modifications on a given peptide was allowed during the search. The in-house program Xl-tools was used to identify, validate, and summarize cross-linked peptides based on MS^*n*^ data and database searching results^51^. Following integration of MS^*n*^ data, no cross-links involving decoy proteins were identified. Only cross-linked peptides that were identified in all three biological replicates are reported.

### Electron microscopy grid preparation and image acquisition

For cryo-EM data collection, 4 μl of purified TcdB^core^–CSPG4^mini^ complex was applied at a concentration of ~0.2 mg/ml to glow-discharged holey carbon grids (Quantifoil Grid R2/2 Cu 200 mesh). The grids were blotted for 1.5 s using an FEI Vitrobot plunger at 10 °C and 100% humidity, and then plunge-frozen in liquid ethane cooled by liquid nitrogen. Two datasets were collected from two grids using similar parameters. For both data collections, cryo-EM imaging was performed on a Titan Krios electron microscope equipped with a Gatan K3 direct electron detector and a Gatan Image Filter using slit width of 20 eV. The microscope was operated at 300 keV accelerating voltage, at a magnification of 105 kX in super-resolution mode resulting in a pixel size of 0.415 Å. All images were automatically recorded using SerialEM^52^. For the first data set, movies were obtained at an accumulated dose of 40 e-/Å^2^ with defocus ranging from −1.2 to −2.2 μm. For the second data set, movies were obtained at an accumulated dose of 46 e-/Å^2^ with defocus ranging from −1.2 to −2.2 μm. The total exposure time was 2.3 s over 66 frames per movie stack. We noticed that the first data set had preferred orientation problem during data processing. Therefore, we collected a second data set using a grid with a thicker ice layer, which yielded more particles with better orientations.

### Image processing and structure determination

All acquired movies underwent patch motion correction and patch CTF estimation in cryoSPARC v2. Particles were auto-picked using blob picker in cryoSPARC. The following 2D, 3D classifications, and refinements were all performed in cryoSPARC^53^. For each of the two datasets, we first extracted particles with a box size of 896 × 896 pixels and bin the data by 4. After rounds of 2D classification, we obtained 559,247 good particles by merging the two datasets, which were used for ab initio reconstruction into five classes, following by further heterogeneous refinement. We chose one of the best classes with clear features for homogeneous refinement. After non-uniform refinement followed by local refinement with a mask, we got a 3.37 Å resolution map, which showed the overall shape of the TcdB^core^–CSPG4^mini^ complex. Similarly, we also tried to use a box size of 576 × 576 pixels and bin the data by 3. After rounds of 2D classification, we obtained 560,946 good particles by merging the two datasets, which were used for ab initio reconstruction into five classes, following by further heterogeneous refinement. We chose one of the best classes with clear features and best resolution for homogeneous refinement. After non-uniform refinement followed by local refinement with a tight mask to omit the highly flexible and low resolution region, we obtained a 3.17 Å resolution density map, which was sharpened using local sharpening in Phenix^54^. Using the full-length TcdB structure as an input model, we were able to build a model for the TcdB^core^–CSPG4^mini^ complex using Phenix^54^. This initial structure model was used for iterative manual building in Coot and real space refinement in Phenix^54,55^. Figures were generated using PyMOL (Schrödinger) and UCSF chimera^56^.

### Dynamic light scattering assay

Dynamic light scattering (DLS) was performed using a Malvern Instruments Zetasizer Nano series instrument and data were analyzed using Zetasizer Version 7.12 software. In total, 100 µl of the TcdB^core^–CSPG4^mini^ complex at 0.1 mg/ml was assayed at 25 °C. A representative DLS profile from three similar results was reported.

### Bio-layer interferometry (BLI) assays

The binding affinities between TcdB and Repeat1 were measured by BLI assay using an OctetRED96 (ForteBio). Prior to use, biosensors were soaked in the assay buffer (20 mM HEPES, 400 mM NaCl, pH 7.5, 10 mM CaCl_2_, 0.1% Tween-20, 0.5% BSA) for at least 10 min. Briefly, Repeat1-Fc (50 nM) was immobilized onto capture biosensors (Dip and Read Anti-hIgG-Fc, ForteBio) and balanced with the assay buffer. The biosensors were then exposed to different concentrations of TcdB1 or TcdB2, followed by the dissociation in the same assay buffer. Binding affinities (*K*_d_) were calculated using the 1:1 binding model by ForteBio Data analysis HT 10.0.

To analyze the competition between bezlotoxumab and CSPG4 on binding to TcdB, the His-tagged Repeat1 (200 nM), which was biotinylated using EZ-Link NHS-PEG4-Biotin (Thermo Fisher Scientific) at pH 6.5^10^, was immobilized onto capture biosensors (Dip and Read Streptavidin, ForteBio) and balanced with the assay buffer. The biosensors were first exposed to TcdB1 or TcdB2 (200 nM), respectively, followed by balanced with the assay buffer. The biosensors were then applied to bezlotoxumab (200 nM), followed by the dissociation in the assay buffer. Reversely, bezlotoxumab (200 nM) was immobilized onto capture biosensors (Dip and Read Anti-hIgG-Fc, ForteBio) and balanced with the assay buffer. The biosensors were first exposed to TcdB1 or TcdB2 (200 nM), respectively, followed by balanced with the assay buffer. The biosensors were then applied to CSPG4^mini^ (200 nM), followed by the dissociation in the assay buffer.

### Protein melting assay and size-exclusion chromatography

The thermal stability of TcdB1 variants was measured using a fluorescence-based thermal shift assay on a StepOne real-time PCR machine (Life Technologies). Each protein (~0.5 mg/ml) was mixed with the fluorescent dye SYPRO Orange (Sigma-Aldrich) and heated from 25 to 95 °C in a linear ramp. The midpoint of the protein melting curve (*T*_m_) was determined using the analysis software provided by the instrument manufacturer. Data obtained from three independent experiments were averaged to generate the bar graph. The folding of Repeat1-Fc variants was verified by Superdex-200 size-exclusion chromatography.

### Pull-down assays

For the structure-based mutagenesis studies, interactions between TcdB and CSPG4 were examined using pull-down assays using Protein A or Strep-Tactin resins in a binding buffer containing 20 mM HEPES, pH 7.5, 150 mM NaCl, 10 mM CaCl_2_, and 0.1% Tween-20. When testing the TcdB variants, Repeat1-Fc was used as the bait and TcdB variants (WT and mutants) were the preys. Repeat1-Fc (45 µg) was preincubated with Protein A resins at room temperature for 1 h, and the unbound protein was washed away using the binding buffer. The resins were then divided into small aliquots and mixed with TcdB variants (~4-fold molar excess over Repeat1-Fc). Pull-down assays were carried out at room temperature for 3 h. The resins were then washed twice, and the bound proteins were released from the resins by boiling in SDS-PAGE loading buffer at 95 °C for 5 min. A similar protocol was used to examine the interactions between Repeat1-Fc variants (preys) and the Twin-Strep tagged TcdB1 (bait) immobilized on Strep-Tactin resins, as well as the simultaneous binding of Repeat1-Fc and CRD2 (preys) to the Twin-Strep tagged TcdB1 (bait). CRD2 was expressed and purified as described previously^24^. Samples were analyzed by SDS-PAGE and Coomassie Blue staining.

The competition between bezlotoxumab and CSPG4 on binding to TcdB was examined by two-step pull-down assays using Protein A or Strep-Tactin resins. In the first set of experiments, bezlotoxumab served as the bait, TcdB1 or TcdB2 was the preys in the first step and CSPG4^mini^ was the prey in the second step. Specifically, bezlotoxumab (40 µg) was preincubated with Protein A resins at 12 °C for 1 h and the unbound protein was washed away. The bezlotoxumab-bound resins were then divided into small aliquots and mixed with ~2-fold molar excess of TcdB1 or TcdB2 and the unbound toxins were washed away after 2 h incubation at 12 °C. Lastly, CSPG4^mini^ (~4-fold molar excess over bezlotoxumab) or the blank binding buffer was added to each tube. After incubation at 12 °C for 2 h, the resins were washed twice and the bound proteins were heating released from the resins at 95 °C for 5 min and further examined by 4–20% SDS-PAGE.

In the second set of experiments, 20 µg of biotin labeled CSPG4^mini^ was used as the bait and preincubated with Strep-Tactin resins at 12 °C for 1 h. The unbound protein was washed away and the CSPG4^mini^-bound resins were then divided into small aliquots. TcdB1 or TcdB2 (~2-fold molar excess over CSPG4^mini^) were the preys in the first step and bezlotoxumab (~4-fold molar excess over CSPG4^mini^) was the prey in the second step. The two-step pull-down assays were carried out using a protocol similar to the one described above.

### *C. difficile* infection assay

All the animal studies were conducted according with ethical regulations under protocols approved by the Institute Animal Care and Use Committee at Boston Children’s Hospital (18-10-3794R). *Clostridioides difficile* infection model has been described previously^57^. C57BL/6 mice were originally purchased from Charles River and a colony was established in the same room hosting CSPG4 KO mice (but two strains were not cohoused in the same cage). CSPG4 KO mice were obtained from Dr. William Stallcup’s lab^36^. Briefly, mice (6–8 weeks, both male and female) were fed with a mixture of antibiotics in water for 3 days (kanamycin (0.4 mg/ml), gentamicin (0.035 mg/ml), colistin (850 U/ml), metronidazole (0.215 mg/ml), and vancomycin (0.045 mg/ml)). The mice were then fed with normal water for 1 day, and intraperitoneally injected (i.p. injection) with a single dose of clindamycin (10 mg/kg). One day after the clindamycin injection, animals were challenged with the PBS control or *C. difficile* spores (1 × 10^5^ or 1 × 10^4^ per mouse) and monitored twice daily for 48 h. Symptoms such as diarrhea, body weight loss, and behavior changes were recorded^35^. Animals were euthanized with CO_2_ asphyxiation when animals were moribund; or animals had weight loss of or greater than 15% body weight. All live mice at 48 h were euthanized to harvest the cecum and colon tissues, which were subjected to either hematoxylin and eosin (H&E) staining for histological score analysis or immunofluorescence staining for Claudin-3.

### Preparation of *C. difficile* spores

Preparation of *C. difficile* spores has been described previously^58^. Briefly, *C. difficile* was recovered from a −80 °C freezer with Brain Heart Infusion medium (Fischer Scientific) plus 5% yeast extract (BD Difco), and cultured for 24 h at 37 °C in an anaerobic chamber until stationary phase. *C. difficile* culture was then spread out on 70:30 plates^59^ with a cotton swab. Spores were harvested and purified with 50% ethanol after 14-day growth and sporulation, and frozen at −80 °C for storage.

### Hematoxylin and eosin (H&E) staining for histology analysis and immunofluorescence staining

H&E staining and immunofluorescence staining have been described previously^24^. Briefly, the cecum or colon tissues were washed with PBS until the fecal contents were removed completely. The tissues were fixed in 10% phosphate buffered formalin for 24 h, embedded in paraffin, and sectioned 6 μm each. Histology analysis was carried out with H&E staining. Stained sections were scored by two observers blinded to experimental groups, based on four criteria including inflammatory cell infiltration, hemorrhagic congestion, epithelial disruption, and submucosal edema on a scale of 0–3 (normal, mild, moderate, or severe). The total histological scores were the addition of scores from the four criteria. Immunofluorescence analysis of Claudin-3 was carried out using rabbit polyclonal anti-Claudin-3 (Abcam, ab15102, 1:100) antibody. The images were taken by Olympus microscopy IX51 (software cellSens standard 1.15) and Zeiss microscopy (software Zen 2.5).

### Cell cytopathic rounding assay

The cytopathic effect (cell rounding) of WT and mutated TcdB was analyzed by standard cell-rounding assay. Briefly, cells were exposed to a gradient of TcdB and TcdB mutants for 6 and 24 h. The phase-contrast images of cells were taken (Olympus IX51, ×10–20 objectives). The numbers of round shaped and normal shaped cells were counted manually. The percentage of round shaped cells was plotted and fitted using the GraphPad Prism software. CR_50_ is defined as the toxin concentration that induces 50% of cells to be rounded in 24 h. Data were represented as mean ± sd from three independent biological replicates.

### Cell surface binding assay

Binding of WT and mutated TcdB to cells was analyzed by the cell surface binding assay^21^. Briefly, cells were exposed to TcdB (10 nM) or TcdB mutants (10 nM) for 10 min at room temperature. Cells were washed three times with PBS and lysed with RIPA buffer (50 mM Tris, 1% NP40, 150 mM NaCl, 0.5% sodium deoxycholate, 0.1% SDS, with a protease inhibitor cocktail (Sigma-Aldrich). Cell lysates were centrifuged and supernatants were subjected to western blotting using chicken polyclonal anti-TcdB IgY (List Labs, #754A, 1:2000) and goat anti-chicken IgY H&L (HRP) (Abcam, ab97135, 1:2000) antibodies to examine the binding of TcdB mutants. Chicken polyclonal anti-actin antibody (Aves Labs, ACT-1010, 1:2000) was used for negative control.

### Cecum injection assay

The in vivo toxicity of WT and mutated TcdB was tested by the cecum injection assay^24^. Briefly, mice (CD1, 6–8 weeks, both male and female, purchased from Envigo) were fasted 19 h and then deeply aestheticized with 3% isoflurane. A midline laparotomy was performed, and 100 µl of PBS, TcdB (6 µg), or TcdB mutant (6 µg) was injected across the ileocecal valve into the cecal lumen via an insulin syringe (31G). The incision was closed with absorbable suture (5-0 Vicryl). The cecum was harvested after a 6 h recovery period. Tissues were fixed in 10% formalin, paraffin-embedded, sectioned, and subjected to either H&E staining for histological score analysis or immunofluorescence staining for Claudin-3.

### In vitro protection assay

The in vitro protection efficacy of inhibitors was tested by the cytopathic rounding effect. Briefly, TcdB1 (10 pM) or TcdB2 (100 pM) were preincubated with two-fold serial-diluted inhibitors in DMEM medium (with 3 mM CaCl_2_) at 37 °C for 2 h. Cells were then exposed to the toxin, or toxin-inhibitor mixture, for the indicated time. The phase-contrast images of cells were taken (Olympus IX51, ×10–20 objectives). The numbers of round shaped and normal shaped cells were counted manually. The percentage of round shaped cells was plotted and fitted using the GraphPad Prism software. Data were represented as mean ± sd from three independent biological replicates.

### In vivo protection assay

The in vivo protection efficacy of inhibitors was tested by the cecum injection assay. Briefly, TcdB1 (6 µg) and TcdB2 (6 µg) were premixed with Repeat1-Fc (30 µg) or bezlotoxumab (52 µg). The PBS control, toxin, toxin with Repeat1-Fc or bezlotoxumab, or the Repeat1-Fc control was injected into the connection part between ileum and cecum, following fasting and anesthesia of CD1 mice. The cecum tissue of animals was harvested after 6-h recovery, and subjected to H&E staining for histological score analysis.

### Colony forming units (CFU) quantification during the infection

The CFU/g feces of *C. difficile* and the TcdB titer/g feces of infected mice were quantified^59^. Briefly, the mice were fed with antibiotic water for 3 days. Regular water was resumed for 1 day, followed with i.p. injection of one dose of clindamycin (10 mg/kg). *C. difficile* spores (1 × 10^4^ per mouse) were administrated via oral gavage 24 h after the clindamycin injection. Feces were collected (at 24, 48, and 72 h after infection), weighted, and frozen at −80 °C immediately until ready to use. For CFU counting, feces were completely dissolved in 500 µl PBS plus 500 µl 95% ethanol and sat for 1 h at room temperature. Dissolved feces were then serial diluted and plated on *C. difficile* selected plates (CHROMID^®^ C. DIFFICILE, BioMérieux). *C. difficile* spores were incubated 24 h at 37 °C anaerobically, and CFU was counted manually and standardized to per gram feces.

### Reporting summary

Further information on research design is available in the Nature Research Reporting Summary linked to this article.

## Supplementary information

Supplementary information

Reporting Summary

## Data Availability

The cryo-EM map and the structural model of the TcdB–CSPG4 complex have been deposited in the Electron Microscopy Data Bank (EMDB) and the Protein Data Bank (PDB) under accession codes EMD-23909 and 7ML7, respectively. Other PDBs used in this paper include: 6OQ5, 6C0B, and 4NP4. Other data supporting the findings of this study are available from the authors upon request. Source data are provided with this paper.

## Source data

Source data
