# Supplementary material for: Structural basis for CSPG4 as a receptor for TcdB and a therapeutic target in Clostridioides difficile infection
Source: Nat Commun. 2021 Jun 18;12:3748. doi: 10.1038/s41467-021-23878-3 (PMC8213806; doi:10.1038/s41467-021-23878-3)
Supplement: Supplementary file 1 — Supplementary information [file 41467_2021_23878_MOESM1_ESM.pdf]

Supplementary Figures

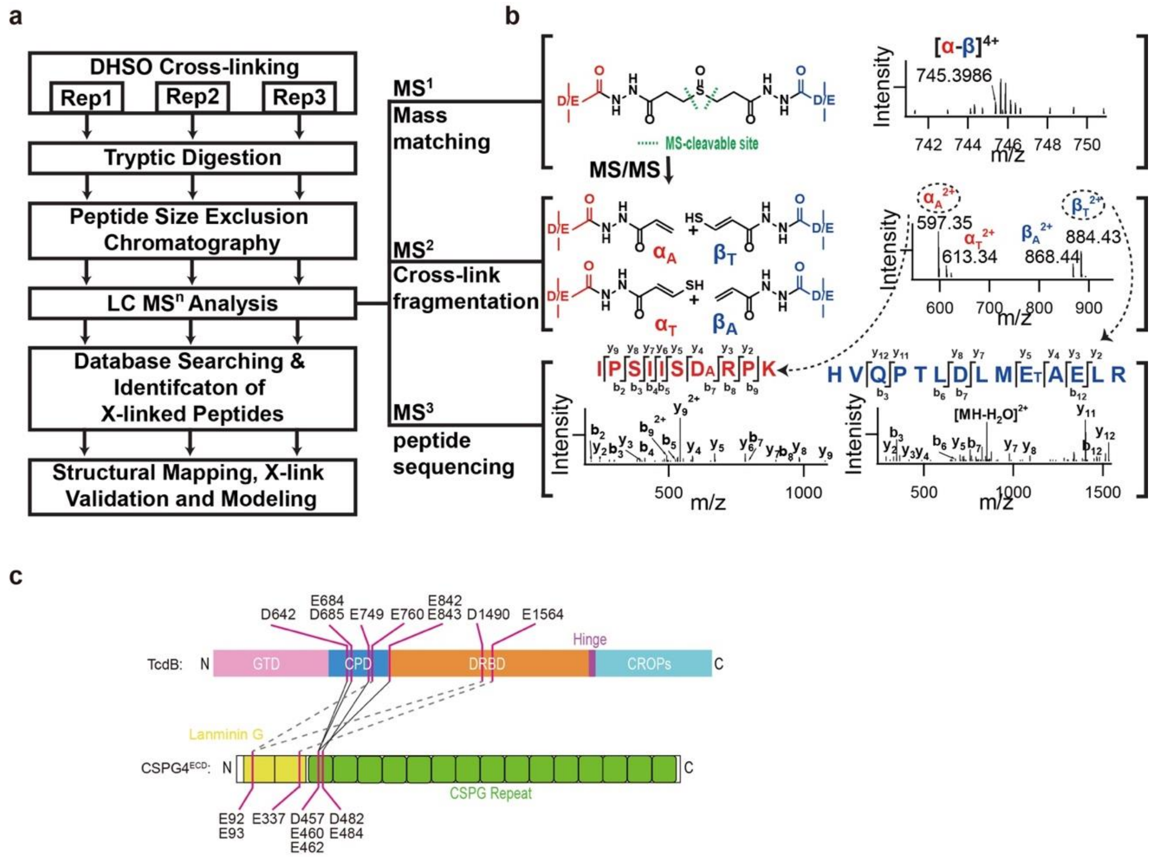

Supplementary Fig. 1 Cross-linking mass spectrometry (XL-MS) studies of the TcdB–CSPG4 complex. a, XL-MS analysis workflow for accurate identification of DHSO cross-linked peptides from 3 replicates (Rep1–3) of cross-linked TcdB–CSPG4 complex. **b**, Representative MS^n identification of a DHSO inter-linked peptide of the TcdB–CSPG4 complex. First, the cross-linked peptide $[\alpha–\beta]^{4+}$ (m/z 745.3986^{4+}) was detected in MS^1. Next, it was selected for MS^2 analysis and yielded two characteristic fragment ion pairs, i.e. α_A/β_T (m/z $597.35^{2+}/884.43^{2+}$) and α_T/β_A (m/z $613.34^{2+}/868.44^{2+}$). Finally, MS^3 analysis of α_A (m/z 597.35^{2+}) identified its sequence as ^{636}IPSIISD$_A$RPK^{645}, in which the aspartic acid residue at position 7 was modified with an alkene moiety. MS^3 analysis of β_T (m/z 884.43^{2+}) identified its sequence as ^{451}HVQPTLDLME$_T$AELR^{464}, in which the glutamic acid residue at position 10 was modified with unsaturated thiol moiety. Thus, the cross-link of TcdB:D642 to CSPG4:E460 was determined. **c**, Illustrations of the identified inter-protein cross-links between CSPG4 and TcdB in the context of the full length proteins. We noted that residue E92 of CSPG4 could be cross-linked to E760 and D1490 that are located in the CPD and DRBD of TcdB, respectively. These two residues are ~97 Å away from each other on TcdB holotoxin, which cannot be simultaneously reached by E92 of CSPG4 via DHSO that has a distance limitation of ~35 Å. This data suggests that the laminin G motifs of CSPG4 adopt flexible conformations and could transiently move within ~35 Å of the CPD or DRBD of TcdB. The linkages between the flexible regions of CSPG4 and TcdB were shown as dashed lines.

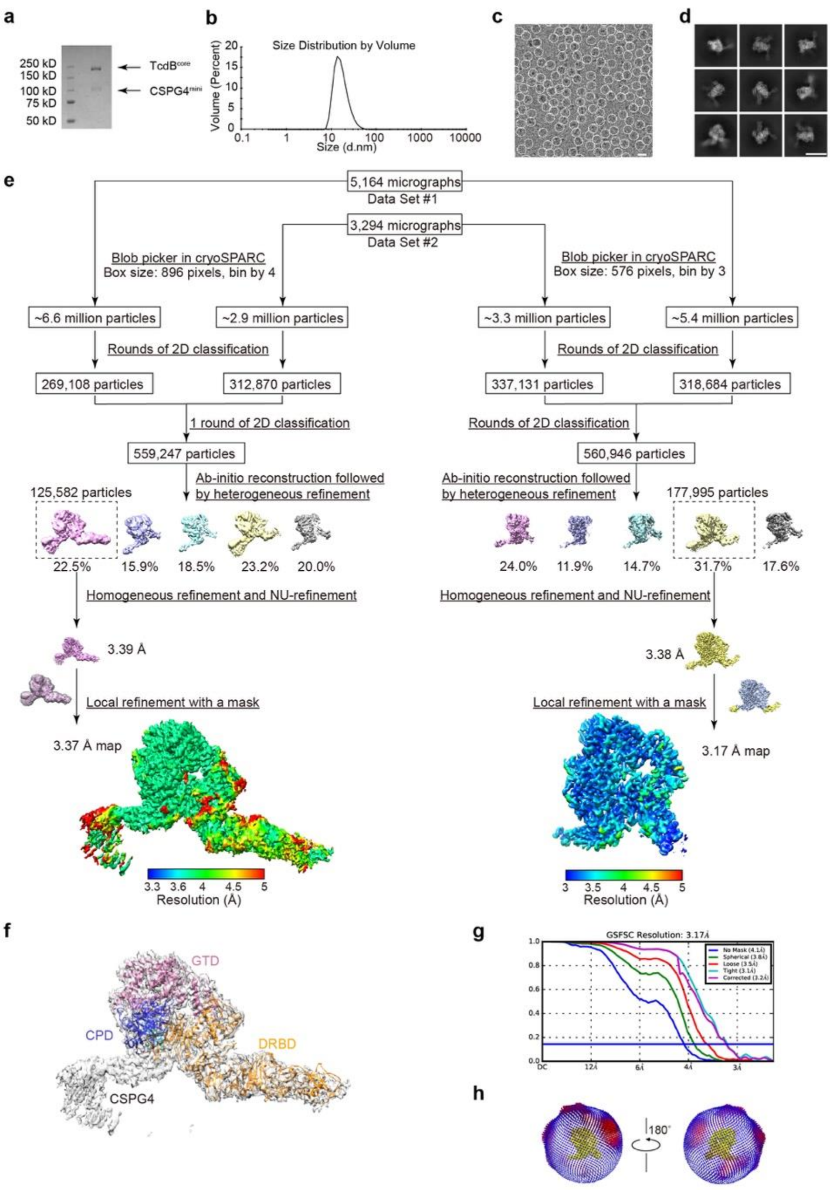

Supplementary Fig. 2 Biochemical characterization and workflow of cryo-EM reconstruction of the TcdB–CSPG4 complex. a-b, The quality of the TcdB^{core}–CSPG4^{mini} complex used for cryo-EM studies was characterized by SDS-PAGE and dynamic light scattering (DLS), a representative result from 3 similar results was reported. **c-d**, An example of a cryo-EM micrograph, the scale bar represents 83 Å (**c**) and 2D classes, the scale bar represents 120 Å (**d**). **e**, Overview of the cryo-EM data processing and structure determination of the TcdB–CSPG4 complex using different box sizes. Overviews of reconstruction of the TcdB–CSPG4 complex are shown in the bottom panels. **f**, TcdB holotoxin (PDB: 6OQ5) was fitted to a 3.37 Å resolution EM map. **g**, Gold-standard Fourier shell correlation (FSC) plots of 3D reconstruction of the 3.17 Å resolution map as calculated in cryoSPARC. **h**, Angular distribution of particles included in the final cryo-EM reconstruction of the 3.17 Å resolution map.

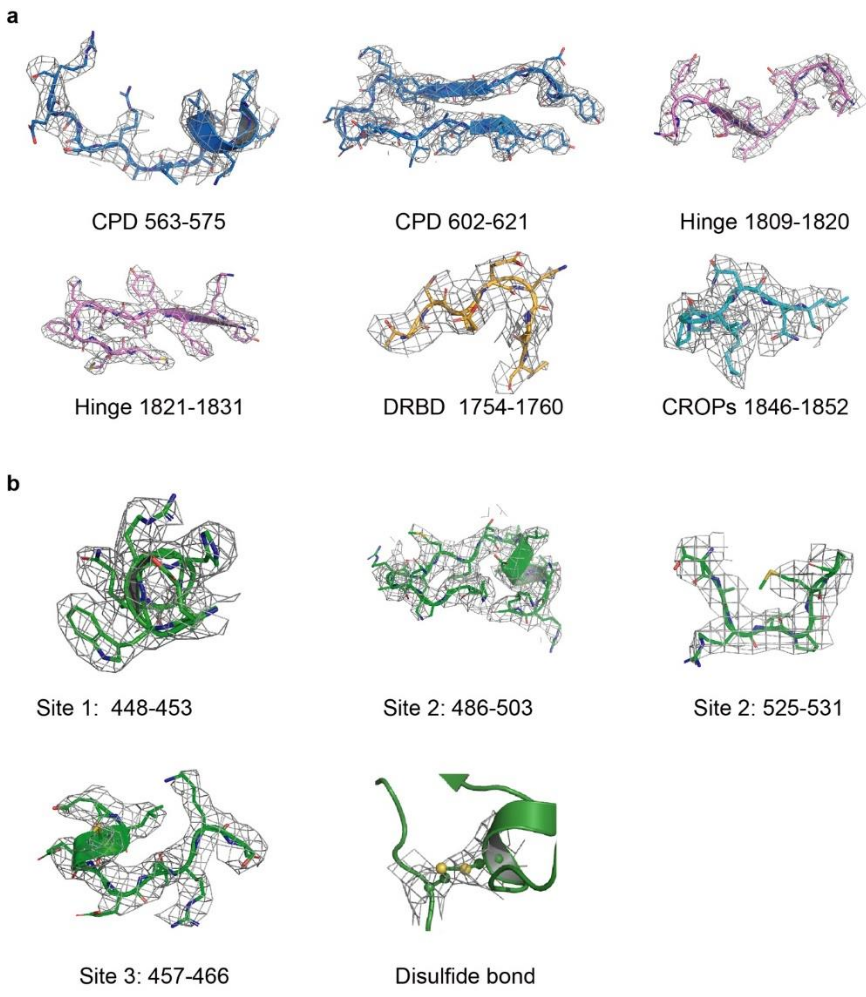

Supplementary Fig. 3 Representative cryo-EM densities of the TcdB–CSPG4 complex at 3.17 Å resolution. Representative cryo-EM densities for TcdB (**a**) and CSPG4 (**b**).

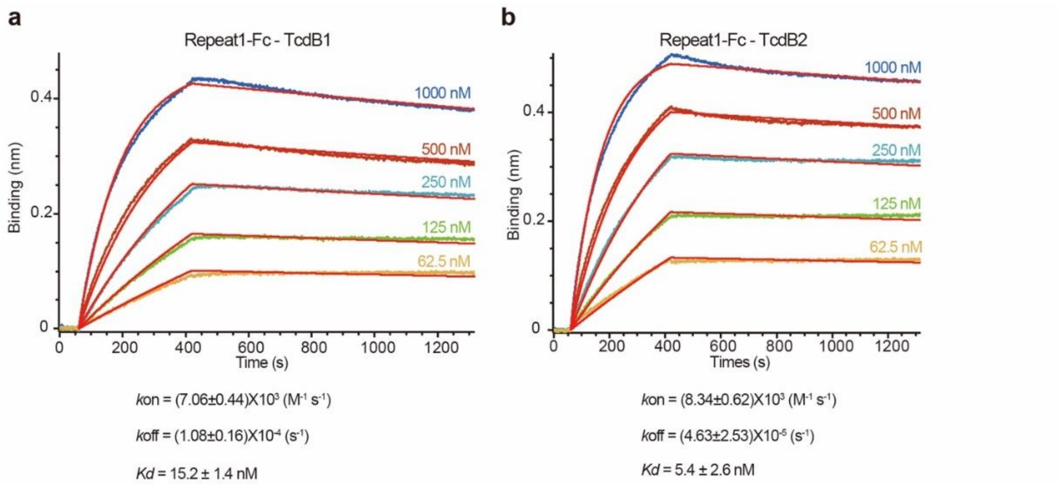

Supplementary Fig. 4 Bio-layer interferometry (BLI) analyses of TcdB1 and TcdB2 binding to CSPG4 Repeat1-Fc. a-b, Representative binding curves with CSPG4 Repeat1-Fc as a ligand immobilized on anti-human IgG Fc capture (AHC) biosensors and TcdB1 or TcdB2 as the analytes. The concentrations of TcdB1 and TcdB2 examined were labeled in each panel. The shown binding analysis results are means ± s.d. from three independent experiments.

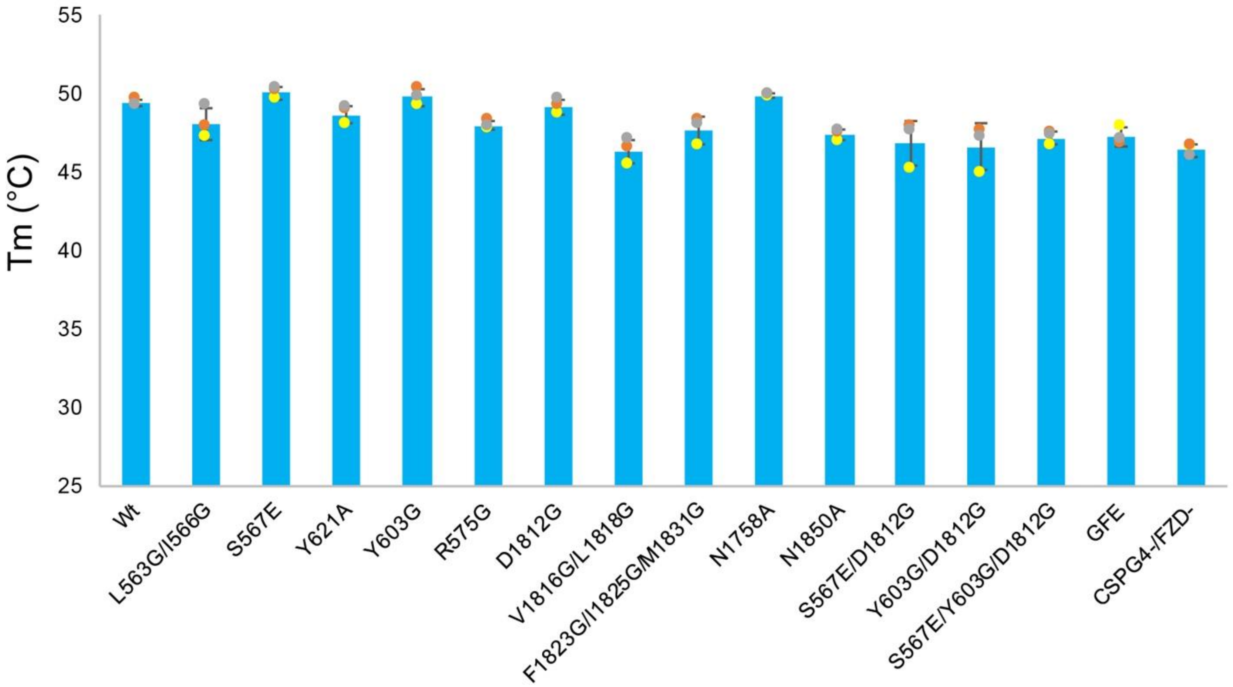

Supplementary Fig. 5 TcdB variants adopt wild-type-like structures. The thermal stability of proteins was measured using a fluorescence-based thermal shift assay on a StepOne real-time PCR system (ThermoFisher). Protein melting was monitored using a hydrophobic dye, SYPRO Orange (Sigma-Aldrich), as the temperature was increased in a linear ramp from 25°C to 95°C. The midpoint of the protein-melting curve (T_m) was determined using the software provided by the instrument manufacturer. The data are presented as means ± s.d. (n=3). All the TcdB1 variants showed T_m values comparable to the wild-type protein, indicating correct protein folding.

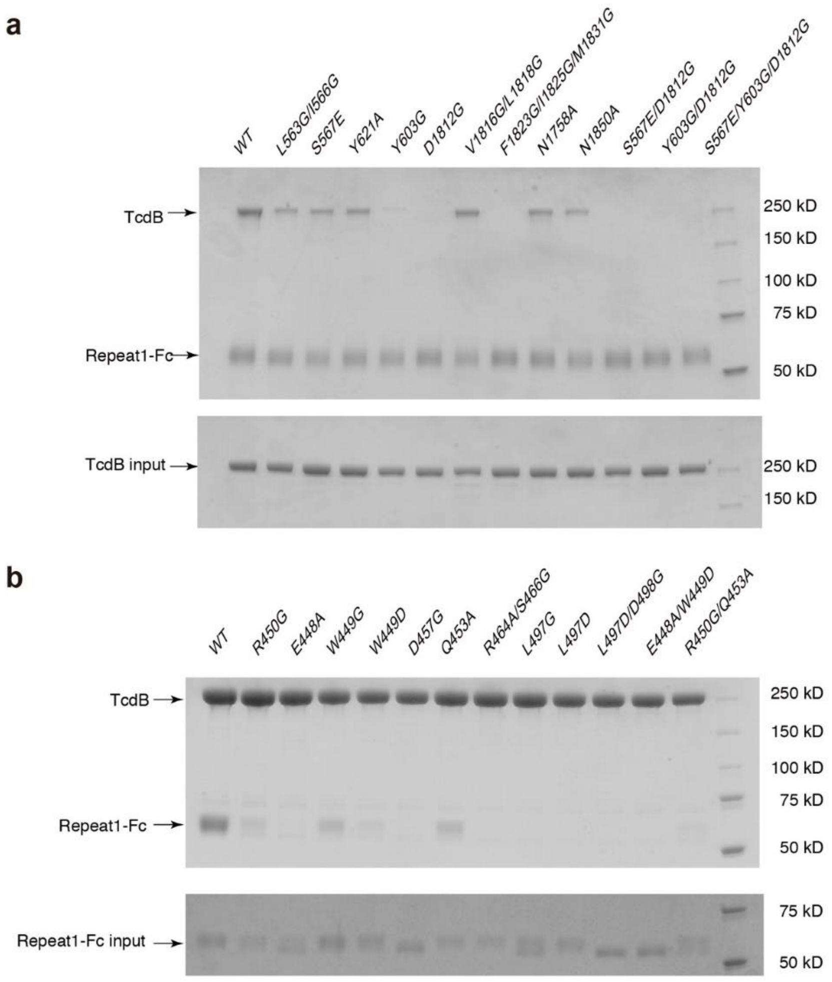

Supplementary Fig. 6 Characterization of the interactions between TcdB and CSPG4 by structure-based mutagenesis. a, The binding of TcdB1 variants to Repeat1-Fc immobilized on Protein A resins was examined using pull-down assays. **b**, The binding of Repeat1-Fc variants to the Twin-strep tagged TcdB1 immobilized on Strep-Tactin resins was examined using pull-down assays. Samples were analyzed by SDS-PAGE and Coomassie Blue staining. The gels are representative of three independent experiments.

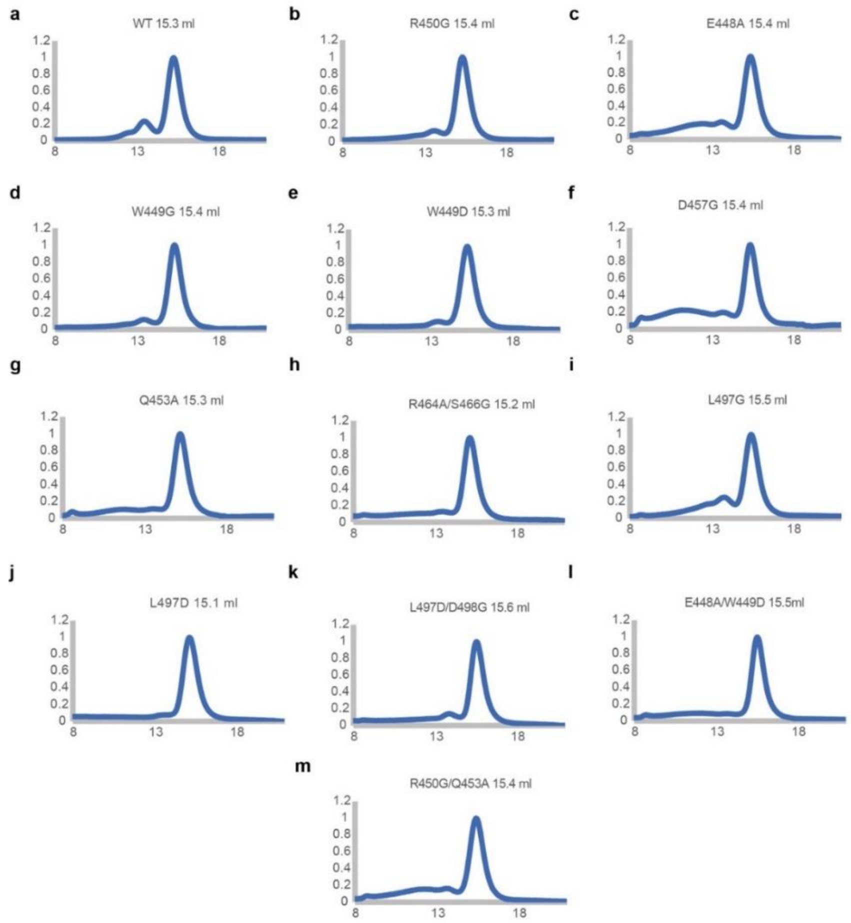

Supplementary Fig. 7 Size-exclusion chromatography analyses of Repeat1-Fc and its variants. a-m, Representative elution profiles of Repeat1-Fc and its variants over a Superdex 200 Increase size-exclusion column, with the horizontal and vertical axes representing the elution volume and the normalized OD_{280} absorbance, respectively. The peak elution volume for each protein is listed.

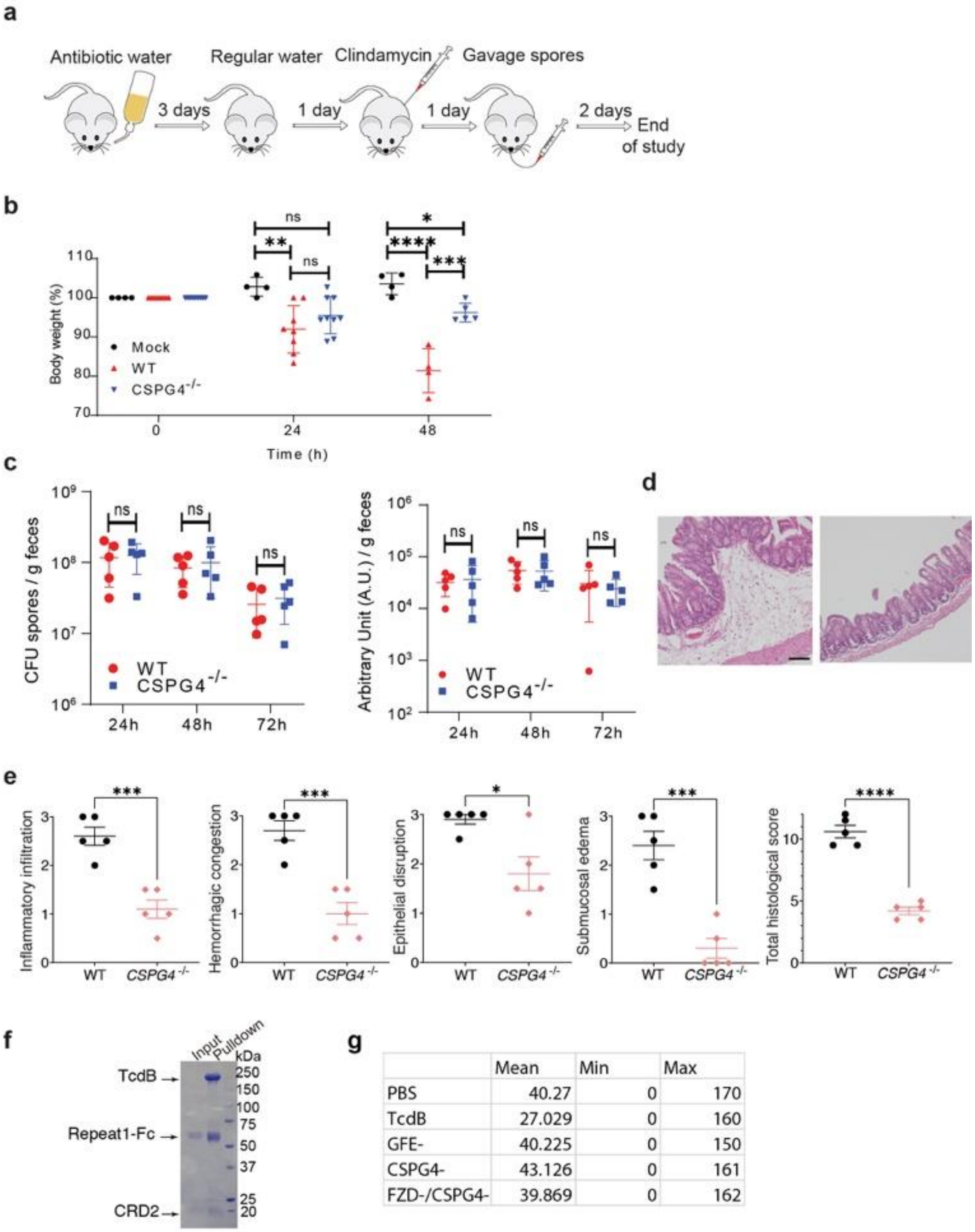

	Mean	Min	Max
PBS	40.27	0	170
TcdB	27.029	0	160
GFE-	40.225	0	150
CSPG4-	43.126	0	161
FZD-/CSPG4-	39.869	0	162

Supplementary Fig. 8 Analysis of *C. difficile* colonization and colon tissue damage in CDI mouse models and cecum injection models. a, Schematic diagram of *C. difficile* infection model. WT and CSPG4$^{-/-}$ mice were fed with antibiotic water for three days before resuming regular water for 24 h. A single dose of clindamycin (10 mg/kg) was administered to mice via intraperitoneal injection (i.p.). *C. difficile* spores (M7404, *tcdA*$^{-}$) and mock (PBS) were administered to mice through oral gavage at 24 h after the injection. Mice were observed for another 48 h. **b**, The WT and CSPG4$^{-/-}$ mice were infected with 1 x 10^5 *C. difficile* spores. Three groups of infection experiment were performed: mock to WT (n=4); M7404, *tcdA*$^{-}$ to WT mice (n=8); and M7404, *tcdA*$^{-}$ to CSPG4$^{-/-}$ mice (n=9). The weight loss of mice was recorded and shown. Error bars indicate

mean ± s.d., p-values by one-way ANOVA: ****: $p \leq 0.0001$, ***: $p \leq 0.001$ **: $p \leq 0.01$, *: $p \leq 0.05$. The p-values of mock vs. *C. difficile* to WT, mock vs. *C. difficile* to CSPG4$^{-/-}$, and *C. difficile* to WT vs. *C. difficile* to CSPG4$^{-/-}$ at 24 h are 0.0061, 0.0624, and 0.3314, at 48 h are <0.0001, 0.0383, and 0.0004. **c-e**, The WT (n = 5) and CSPG4$^{-/-}$ mice (n = 5) were infected with 1 x 10^4 *C. difficile* spores (M7404 *tcdA*$^-$). Feces were collected at 24 h, 48 h, and 72 h, and dissolved in 50% ethanol. The dissolved feces were serial diluted, and the colony-forming unit (CFU) of *C. diff* spores / gram (g) of feces were quantified **(c**, left panel**),** and the toxin titter (arbitrary unit / gram feces) was tested by the cytopathic effects **(c**, right panel**)**. The arbitrary unit was defined as the dilution fold to reach CR_{50}. Error bars indicate mean ± s.d., p-values by t test: ****: $p \leq 0.0001$, ***: $p \leq 0.001$ **: $p \leq 0.01$, *: $p \leq 0.05$. The p-values of 24 h, 48 h, and 72h for CFU are 0.831465, 0.671835, and 0.616704, for arbitrary toxin are 0.786909, 0.926407, and 0.628095. Cecum tissues were harvested at 90 h and subjected to H&E staining (scale bar represents 100 µm. M7404, *tcdA*$^-$ to WT mice n = 5, and M7404, *tcdA*$^-$ to CSPG4$^{-/-}$ mice n = 5) (**d**) and histological analysis (**e**). Error bars indicate mean ± s.d., p-values by t test: ****: $p \leq 0.0001$, ***: $p \leq 0.001$ **: $p \leq 0.01$, *: $p \leq 0.05$. The p-values of inflammation, hemorrhagic congestion, epithelial disruption, submucosal edema, and histological scores are 0.0005, 0.0005, 0.0144, 0.0003, and <0.0001. **f**, Repeat1-Fc and CRD2 (preys) were pull down by the Twin-strep-tagged TcdB1 (bait) immobilized on Strep-Tactin resins. Samples were analyzed by SDS-PAGE and Coomassie Blue staining, and the gel is representative of three independent experiments. **g**, The Claudin-3 intensity of immunostaining shown in Figure 5c was quantified by ImageJ.

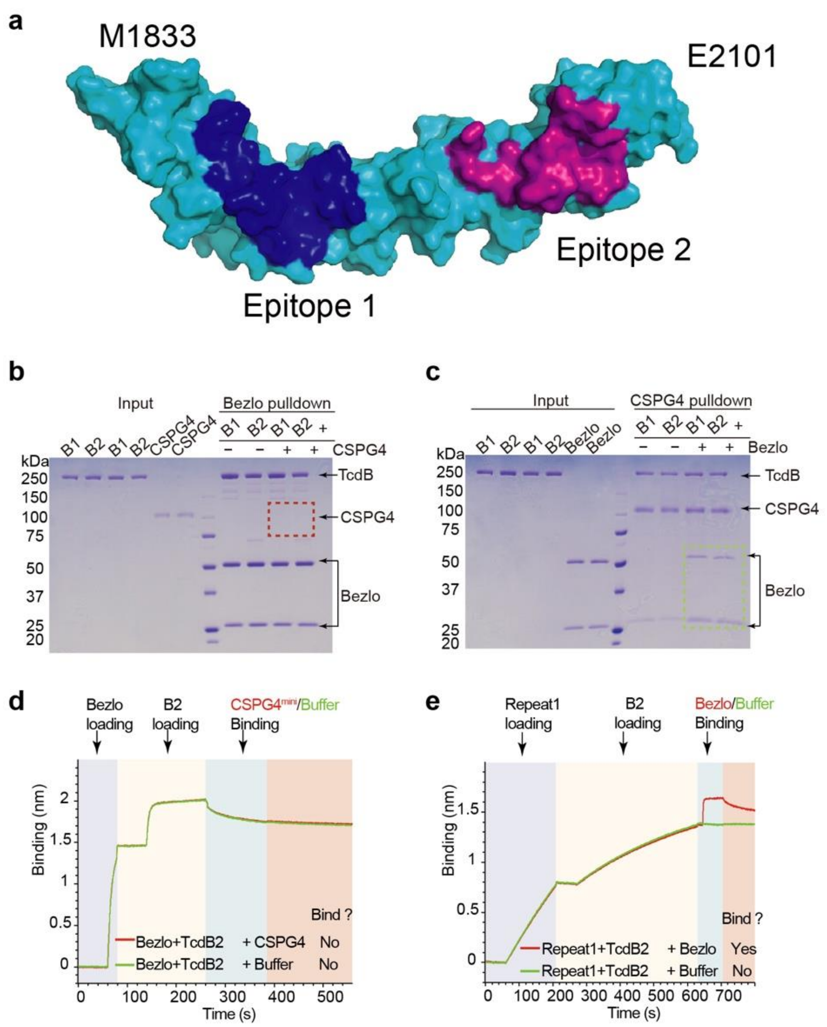

Supplementary Fig. 9 Bezlotoxumab competes with CSPG4 in an allosteric manner. a, The crystal structure of a fragment of TcdB1 consisting of the CROPs I and II (residues 1833–2101) is shown as a surface model, while the epitope-1 and epitope-2 of bezlotoxumab are colored blue and purple, respectively (PDB: 4NP4). **b**, Bezlotoxumab blocks both TcdB1 and TcdB2 from binding to CSPG4^{mini}. In this two-step pull down assay, TcdB1 and TcdB2 were pre-bound to bezlotoxumab immobilized on protein A resins, which were then examined for binding to CSPG4^{mini}. **c**, Bezlotoxumab can still bind to the CSPG4-bound TcdB1 and TcdB2. TcdB1 and TcdB2 were pre-bound to the biotin labeled CSPG4^{mini} immobilized on Strep-Tactin resins, which were then tested for bezlotoxumab binding. **d**, TcdB2 could not bind CSPG4^{mini} when it was pre-bound to the immobilized bezlotoxumab according to BLI assays. **e**, Bezlotoxumab could still bind TcdB2 when it was pre-bound to the immobilized CSPG4 Repeat1. Sequential loading of different proteins to the biosensor is indicated by different background colors.

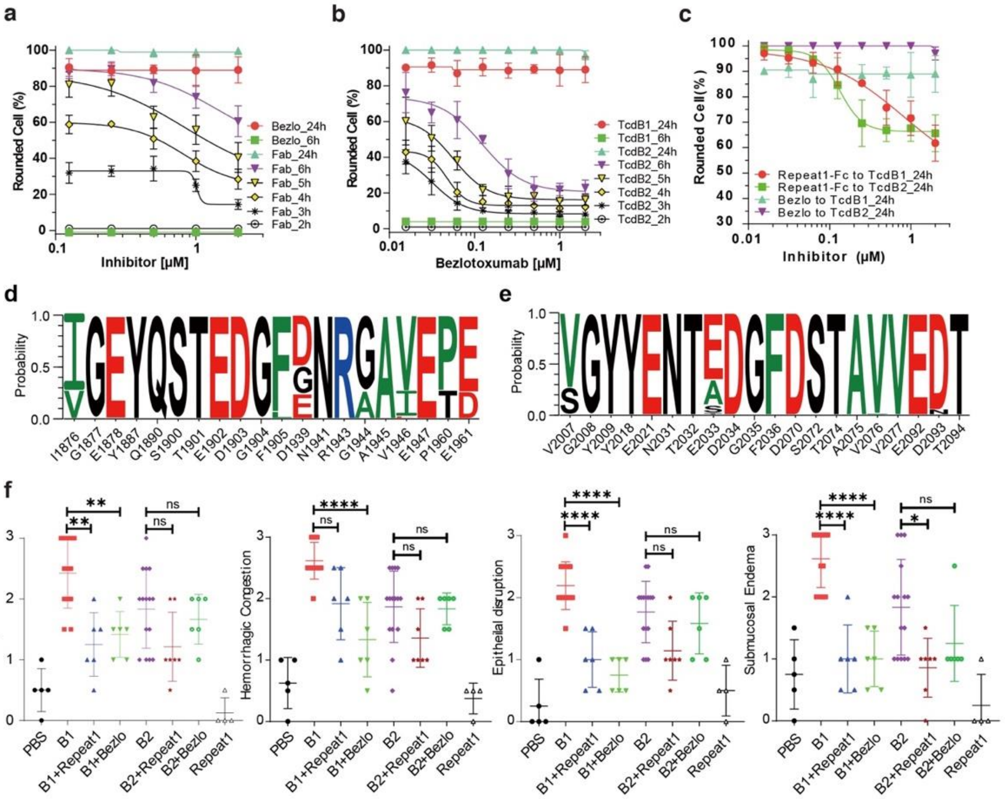

Supplementary Fig. 10 Protection of bezlotoxumab, its Fab fragment, and Repeat1-Fc against TcdB1 and TcdB2. a-c, Protection effects of bezlotoxumab, its Fab fragment, and Repeat1-Fc against TcdB1 and TcdB2 were tested by the cytopathic cell-rounding assay on HeLa cells. HeLa cells were incubated with TcdB1 (10 pM) in the presence of bezlotoxumab or its Fab (**a**); or with TcdB1 (10 pM) or TcdB2 (100 pM) in the presence of bezlotoxumab or Repeat1-Fc (**b-c**). Percentages of rounded cells over time were recorded and plotted. Error bars indicate mean ± s.d. (n=3). **d-e**, Graphical representations of sequence conservation of key amino acids consisting the epitope-1 (**d**) and epitope-2 (**e**) of bezlotoxumab among 206 unique TcdB variants. The height of symbols at each position indicates the relative frequency of each amino acid at that position based on analyses of 206 unique TcdB variants. **f**, The protective effects of Repeat1-Fc and bezlotoxumab against TcdB1 and TcdB2 were examined *in vivo* using the cecum injection assay. TcdB1 (6 µg), TcdB2 (6 µg), TcdB1 or TcdB2 with Repeat1-Fc (30 µg) or bezlotoxumab (52 µg), Repeat1-Fc alone (30 µg), or the PBS control was injected into the cecum of CD1 mice *in vivo*. The cecum tissues were harvested 6 h later and subjected to histological analysis. Error bars indicate mean ± s.d. (PBS n = 5, B1 n = 13, B1 + Repeat1 n = 6, B1 + Bezlo n = 6, B2 n = 15, B2 + Repeat1 n = 7, B2 + Bezlo n = 6, Repeat1 n = 4). p-values by One-way ANOVA: ****: $p \leq 0.0001$, ***: $p \leq 0.001$, **: $p \leq 0.01$, *: $p \leq 0.05$. The p-values of B1 vs. B1 + Repeat 1, B1 vs. B1 + Bezlo, B2 vs. B2 + Repeat 1, B2 vs. B2 + Bezlo for inflammatory cell infiltration are 0.0010, 0.0075, 0.2006, and 0.9979; for hemorrhagic congestion are 0.0707, <0.0001, 0.2771, and >0.9999; for epithelial disruption are <0.0001, <0.0001, 0.0562, and 0.9879; for submucosal edema are <0.0001, <0.0001, 0.0136, and 0.4560.

Supplementary Table 1. Linkages between TcdB and CSPG4 identified by XL-MS.

Linkages	Total IDs	Distance (Å)
TcdB:D642-CSPG4:E460	291	11.6
TcdB:D642-CSPG4:E462	199	16.4
TcdB:E843-CSPG4:E460	62	20.5
TcdB:D685-CSPG4:E460	37	20.1
TcdB:E843-CSPG4:E462	37	22.8
TcdB:D642-CSPG4:D457	36	13.5
TcdB:D685-CSPG4:E462	31	25.2
TcdB:E684-CSPG4:E460	23	23.8
TcdB:E684-CSPG4:E462	20	28.8
TcdB:E842-CSPG4:E460	20	23.1
TcdB:E1564-CSPG4:E337*	19	N/A*
TcdB:E842-CSPG4:E462	16	25.1
TcdB:D1490-CSPG4:E93*	12	N/A*
TcdB:D1490-CSPG4:E92*	8	N/A*
TcdB:E749-CSPG4:D484	7	18.7
TcdB:E749-CSPG4:E482	6	23.4
TcdB:E843-CSPG4:D457	4	24.3
TcdB:E760-CSPG4:E92*	3	N/A*

* These linkages have no known distance as the structure of the N-terminal laminin G motifs have not been determined due to high flexibility.

Supplementary Table 2. Characterization of various TcdB and CSPG4 truncations.

TcdB Fragment	*E. coli* Expression	CSPG4 Binding *	CSPG4 Fragment	HEK293F Expression	TcdB Binding *
1–2366	Yes	Yes	30–2204	Yes	Yes
1–2099	Yes	Yes	30–2148	No	N/A
1–1967	Yes	Yes	30–2028	No	N/A
			30–1465	No	N/A
			30–764	Yes	Yes
			410–551	Yes	Yes
			420–645	No	N/A

* The binding between TcdB and CSPG4 fragments was examined by pull down assays.

Supplementary Table 3. Cryo-EM data collection, refinement, and validation statistics.

	TcdB^{core}–CSPG4^{mini} (EMDB: EMD-23909) (PDB: 7ML7)	
**Data collection and processing**	Data Set #1	Data Set #2
Magnification	105 K	105 K
Voltage (kV)	300	300
Electron exposure (e–/Å^2)	40	46
Defocus range (μm)	-1.2 to -2.2	-1.2 to -2.2
Pixel size (Å)	0.415	0.415
Symmetry imposed	C1	C1
Initial particle images (no.)	5,425,209	3,292,851
Final particle images (no.)	177,995 (Combined Data Sets)	
Map resolution (Å)	3.17	
FSC threshold	0.143	
**Refinement**		
Initial model used (PDB code)	6OQ5	
Model resolution (Å)	3.4	
FSC threshold	0.5	
Map sharpening *B* factor (Å^2)	-55.2	
Model composition		
Non-hydrogen atoms	10,913	
Protein residues	1,354	
Ligands(Zn)	1	
B factors (Å^2)		
Protein	63.26	
Ligand	81.76	
R.m.s. deviations		
Bond lengths (Å)	0.004	
Bond angles (°)	0.743	
Validation		
MolProbity score	1.80	
Clashscore	5.73	
Poor rotamers (%)	0.48	
Ramachandran plot		
Favored (%)	92.10	
Allowed (%)	7.82	
Disallowed (%)	0.07	

Supplementary Table 4. Protein-protein interactions in the TcdB–CSPG4 complex.

	TcdB residues	CSPG4 residues	Type of interaction
CPD – Site 1 interaction	E564	R450	SB
	S567	E448	HB
	L563<br>I566<br>S567<br>Y621<br>Y603<br>P602	W449<br>D457	vdW
	Y621	Q453	HB
Hinge – Site 2 interaction	V1816<br>L1818<br>Y1819<br>F1823<br>I1825<br>M1831	S466<br>L497<br>T495<br>M493<br>P486<br>K503<br>N501	vdW
	Y1819	D498<br>T495 (mc)	HB<br>HB
	N1850	K503	HB
CPD-DRBD-Hinge – Site 3 interaction	R575<br>S573<br>Y1811	M459	vdW
	R575	D457	SB
	T1754 (mc)<br>I1809 (mc)	R464	HB<br>HB
	N1758<br>N1758 (mc)	R527	HB<br>HB
	D1812	S466<br>S466 (mc)	HB<br>HB

"SB", "vdW", and "HB" stand for salt bridge, van der Waals interaction, and hydrogen bond, respectively. "mc" indicates the main-chain-mediated contacts, and all the other contacts are mediated by side-chain atoms.

Supplementary Table 5. List of primers.

	**Forward primer (5'->3')**	**Reverse primer (5'->3')**
$TcdB^{core}$	GGGAATTCCATATGTCTGCTTGGTCTCACCCACAATTCG	CCGCTCGAGTTTGAAAGCCTTGCCGGTTTCCGGGC
Full length TcdB1	CATGCCATGGGCTCTGCTTGGTCTCACCCACAATTCG	CCGCTCGAGTTAGTGATGATGATGATGATGTTCAG
$CSPG4^{mini}$	CGCGGATCCGCTTCCTTCTTCGGTGAGAACCACC	TTCTTGGACCGGTTACTGCACCTCCAGGGCCAGGTTCTCC
Repeat1-Fc	CGCGGATCCGAGCTGCCTGAGCCATGCGTGCCTG	CTAGTCTAGAGTCATTGACAGGGTTGACCTGGATG
$CSPG4^{ECD}$	CCGGAATTCGCTTCCTTCTTCGGTGAGAACCACC	CTAGTCTAGAGCTGGATGCCATGGGGCCTGGCTCG
His-Repeat1	CGCGGATCCGAGCTGCCTGAGCCATGCGTGCCTG	CTAGTCTAGACTAGTCATTGACAGGGTTGACCTGGATG
Bezlo LC	AAAAGGCCTGAGATTGTGCTGACACAGTCCCCCG	CTAGTCTAGATTAGCATTCTCCTCTATTGAAGCTCTTGGTC
Bezlo HC	AAAAGGCCTGAGGTGCAGCTCGTGCAGAGCGGCG	CTAGTCTAGAGCAGGACTTGGGCTCCACCCTCTTATCG
Bezlo V_H-C_H1	AAAAGGCCTGAGGTGCAGCTCGTGCAGAGCGGCG	CTAGTCTAGATTAGCAGGACTTGGGCTCCACCCTCTTATCG
