# Supplementary material for: Structural basis for CSPG4 as a receptor for TcdB and a therapeutic target in Clostridioides difficile infection
Source: Nat Commun. 2021 Jun 18;12:3748. doi: 10.1038/s41467-021-23878-3 (PMC8213806; doi:10.1038/s41467-021-23878-3)
Supplement: Supplementary file 2 — Reporting Summary [file 41467_2021_23878_MOESM2_ESM.pdf]

nature research

Corresponding author(s): Rongsheng Jin & Min Dong

Last updated by author(s): May 12, 2021

Reporting Summary

Nature Research wishes to improve the reproducibility of the work that we publish. This form provides structure for consistency and transparency in reporting. For further information on Nature Research policies, see our Editorial Policies and the Editorial Policy Checklist.

Statistics

For all statistical analyses, confirm that the following items are present in the figure legend, table legend, main text, or Methods section.

n/a	Confirmed	
☐	☒	The exact sample size (*n*) for each experimental group/condition, given as a discrete number and unit of measurement
☐	☒	A statement on whether measurements were taken from distinct samples or whether the same sample was measured repeatedly
☐	☒	The statistical test(s) used AND whether they are one- or two-sided<br>*Only common tests should be described solely by name; describe more complex techniques in the Methods section.*
☒	☐	A description of all covariates tested
☒	☐	A description of any assumptions or corrections, such as tests of normality and adjustment for multiple comparisons
☐	☒	A full description of the statistical parameters including central tendency (e.g. means) or other basic estimates (e.g. regression coefficient) AND variation (e.g. standard deviation) or associated estimates of uncertainty (e.g. confidence intervals)
☐	☒	For null hypothesis testing, the test statistic (e.g. *F*, *t*, *r*) with confidence intervals, effect sizes, degrees of freedom and *P* value noted<br>*Give P values as exact values whenever suitable.*
☒	☐	For Bayesian analysis, information on the choice of priors and Markov chain Monte Carlo settings
☒	☐	For hierarchical and complex designs, identification of the appropriate level for tests and full reporting of outcomes
☒	☐	Estimates of effect sizes (e.g. Cohen's *d*, Pearson's *r*), indicating how they were calculated

Our web collection on statistics for biologists contains articles on many of the points above.

Software and code

Policy information about availability of computer code

Data collection	CellSens Imaging v1.15, Zeiss Zen v2.5, SerialEM was used to collect the Cryo-EM images
Data analysis	Graphpad Prism v8.0, Adobe Illustrator v2.0, cryoSPARC v2.14.2 was used to process the data and to generate all the maps, UCSF Chimera 1.14 and Pymol 2.0.7 were used to analyze the maps and generate figures, Coot v.0.8.9.2 and Phenix v1.18.2 were used to build, refine and evaluate all the atomic models, ForteBio Data analysis HT 10.0 was used to process BLI data.

For manuscripts utilizing custom algorithms or software that are central to the research but not yet described in published literature, software must be made available to editors and reviewers. We strongly encourage code deposition in a community repository (e.g. GitHub). See the Nature Research guidelines for submitting code & software for further information.

Data

Policy information about availability of data

All manuscripts must include a data availability statement. This statement should provide the following information, where applicable:

- Accession codes, unique identifiers, or web links for publicly available datasets
- A list of figures that have associated raw data
- A description of any restrictions on data availability

The cryo-EM map of the TcdB–CSPG4 complex was deposited to the EMDB: EMD-23909 and the structural model to the PDB: 7ML7. Other PDBs used in this paper include: 6OQ5 (https://www.rcsb.org/structure/6oq5), 6C0B (https://www.rcsb.org/structure/6c0b) , and 4NP4 (https://www.rcsb.org/structure/4np4). Other data supporting the findings of this study are available from the authors upon request.

Field-specific reporting

Please select the one below that is the best fit for your research. If you are not sure, read the appropriate sections before making your selection.

☒ Life sciences ☐ Behavioural & social sciences ☐ Ecological, evolutionary & environmental sciences

For a reference copy of the document with all sections, see nature.com/documents/nr-reporting-summary-flat.pdf

Life sciences study design

All studies must disclose on these points even when the disclosure is negative.

Sample size	For high dose C. difficile spore infection, WT mice infected with mock (n=4), WT mice infected with C. difficile spores (n=8), CSPG4-/- mice infected with C. difficile spores (n=9).<br><br>For low dose C. difficile spore infection, WT mice infected with C. difficile spores (n=5), CSPG4-/- mice infected with C. difficile spores (n=5).<br><br>For cecum injection assay, WT mice injected with PBS (m=4), WT mice injected with TcdB (n=5), WT mice injected with TcdB_GFE (n=5), WT mice injected with TcdB_CSPG4 (n=5), WT mice injected with TcdB_FZD_CSPG4 (n=5).<br><br>For protective efficacy of inhibitors: PBS n = 5, B1 n = 13, B1 + Repeat1 n = 6, B1 + Bezlo n = 6, B2 n = 15, B2 + Repeat1 n= 7, B2 + Bezlo n = 6, Repeat1 n = 4<br><br>Sample size was determined based on authors' past experience and what is commonly accepted sample size number in the literature. Each sample size was selected so that a reasonable scientist would conclude that the size is sufficient to draw a statistical conclusion. At least two biological replicates were carried out.
Data exclusions	Data were not excluded from analysis
Replication	All experiments were replicated at least twice. All attempts at replication were successful.
Randomization	Samples were allocated into experimental groups randomly.
Blinding	Stained sections were scored by observers blinded to experimental groups. Blinding was not performed for other experiments, as virtually all the data are quantitative and not easily subject to operator bias.

Reporting for specific materials, systems and methods

We require information from authors about some types of materials, experimental systems and methods used in many studies. Here, indicate whether each material, system or method listed is relevant to your study. If you are not sure if a list item applies to your research, read the appropriate section before selecting a response.

Materials & experimental systems

n/a	Involved in the study	
☐	☒	Antibodies
☐	☒	Eukaryotic cell lines
☒	☐	Palaeontology and archaeology
☐	☒	Animals and other organisms
☒	☐	Human research participants
☒	☐	Clinical data
☒	☐	Dual use research of concern

Methods

n/a	Involved in the study	
☒	☐	ChIP-seq
☒	☐	Flow cytometry
☒	☐	MRI-based neuroimaging

Antibodies

Antibodies used	Chicken polyclonal anti-Actin Aves Labs ACT-1010<br>Horseradish peroxidase chicken anti-mouse IgG Aves Labs IMH-1010<br>Anti-Clostridium difficile Toxin B (Chicken IgY) List Labs 754A<br>Goat Anti-Chicken IgY H&L (HRP) abcam ab97135<br>Anti-Claudin 3 antibody abcam ab15102<br>bezlotoxumab was expressed and purified in the Jin lab
Validation	All antibodies were validated in previous experiments and results were published in Peng, et al, Science, 2018, and Liang, et al, Nature, 2016

Eukaryotic cell lines

Policy information about cell lines

Cell line source(s)	Hela-Cas9 and Hela CSPG4-/- was made in Min Dong's lab<br>FreeStyle™ 293-F Cells (ThermoFisher, R79007)
Authentication	Hela-Cas9 and Hela CSPG4-/- was made in Min Dong's lab, the authentication was published in previous publication Liang, et al, 2016.
Mycoplasma contamination	Cell lines were not tested for mycoplasma contamination but no indication of contamination was observed.
Commonly misidentified lines<br>(See ICLAC register)	No commonly misidentified cell lines were used.

Animals and other organisms

Policy information about studies involving animals; ARRIVE guidelines recommended for reporting animal research

Laboratory animals	Adult CD-1 mice (6- weeks, both male and female, Charles River #022), and adult C56B6/L (6- weeks, both male and female, Jackson Laboratory #000664) were used in this study. All mice were housed in a negative pressure room with temperature 71 F +/- 3 F, light cycle 7:00 to 7:00, and humidity 35% - 70% +/- 5%.
Wild animals	No wild animals were used in the study.
Field-collected samples	No field collected samples were used in the study.
Ethics oversight	The mouse work was performed under the study protocol 18-10-3794R, as approved by the Boston Children's Hospital Institutional Animal Care and Use Committee.

Note that full information on the approval of the study protocol must also be provided in the manuscript.
